# Regimen on *Dnaja3* haploinsufficiency mediated sarcopenic obesity with imbalanced mitochondrial homeostasis and lipid metabolism

**DOI:** 10.1002/jcsm.13549

**Published:** 2024-08-12

**Authors:** Yu‐Ning Fann, Wan‐Huai Teo, Hsin‐Chen Lee, Chen‐Chung Liao, Yeou‐Guang Tsay, Tung‐Fu Huang, Jeng‐Fan Lo

**Affiliations:** ^1^ Institute of Pharmacology, College of Medicine National Yang Ming Chiao Tung University Taipei Taiwan; ^2^ Institute of Oral Biology, College of Dentistry National Yang Ming Chiao Tung University Taipei Taiwan; ^3^ Department of Pharmacy, College of Pharmaceutical Sciences National Yang Ming Chiao Tung University Taipei Taiwan; ^4^ Mass Spectrometry Facility, Instrumentation Resource Center National Yang Ming Chiao Tung University Taipei Taiwan; ^5^ Cancer Progression Research Center National Yang Ming Chiao Tung University Taipei Taiwan; ^6^ Institute of Biochemistry and Molecular Biology, College of Life Science National Yang Ming Chiao Tung University Taipei Taiwan; ^7^ School of Medicine, College of Medicine National Yang Ming Chiao Tung University Taipei Taiwan; ^8^ Department of Orthopedics and Traumatology Taipei Veterans General Hospital Taipei Taiwan; ^9^ Department of Dentistry, College of Dentistry National Yang Ming Chiao Tung University Taipei Taiwan; ^10^ Department of Dentistry Taipei Veterans General Hospital Taipei Taiwan

**Keywords:** DNAJA3, Lipid metabolism, Mitochondrial homeostasis, Sarcopenic obesity, Skeletal muscle homeostasis

## Abstract

**Background:**

Sarcopenic obesity is characterized by excess fat mass and diminished muscular mass/function. DNAJA3, a mitochondrial co‐chaperone protein, plays a crucial role in skeletal muscle development. GMI, an immunomodulatory protein, promotes myogenic differentiation through DNAJA3 activation. This study aims to elucidate the physiological effects of muscular *Dnaja3* haploinsufficiency on mitochondrial dysfunction and dysregulated lipid metabolism and to assess the efficacy of GMI in rescuing sarcopenic obesity both *in vitro* and *in vivo*.

**Methods:**

We generated mouse strain with *Dnaja3* heterozygosity (*HSA‐Dnaja3* ^*f/+*^) specifically in skeletal muscle. The body weight, body composition, and locomotor activity of WT and *HSA‐Dnaja3* ^*f/+*^ mice were examined. The isolated skeletal muscles and primary myoblasts from the WT and *HSA‐Dnaja3* ^*f/+*^ mice, at young or old age, were utilized to study the molecular mechanisms, mitochondrial respiration and ROS level, mitochondrial proteomes, and serological analyses, respectively. To evaluate the therapeutic efficacy of GMI, both short‐term and long‐term GMI treatment were administrated intraperitoneally to the *HSA‐Dnaja3* ^*f/+*^ young (4 weeks old) or adult (3 months old) mice for a duration of either 1 or 6 months, respectively.

**Results:**

Muscular *Dnaja3* heterozygosity resulted in impaired locomotor activity (*P* < 0.05), reduced muscular cross‐sectional area (*P* < 0.0001), and up‐regulation of lipogenesis (ACC2) and pro‐inflammation (STAT3) in skeletal muscles (*P* < 0.05). Primary myoblasts from the *HSA‐Dnaja3* ^*f/+*^ mice displayed impaired mitochondrial respiration (*P* < 0.01) and imbalanced mitochondrial ROS levels. A systemic proteomic analysis of the purified mitochondria from the primary myoblasts was conducted to show the abnormalities in mitochondrial function and fatty acid metabolism (*P* < 0.0001). At age of 13 to 14 months, the *HSA‐Dnaja3* ^*f/+*^ mice displayed increased body fat mass (*P* < 0.001), reduced fat‐free mass (*P* < 0.01), and impaired glucose and insulin tolerance (*P* < 0.01). The short‐term GMI treatment improved locomotor activity (*P* < 0.01) and down‐regulated the protein levels of STAT3 (*P* < 0.05), ACC2, and mitochondrial respiratory complex III (UQCRC2) (*P* < 0.01) via DNAJA3 activation. The long‐term GMI treatment ameliorated fat mass accumulation, glucose intolerance, and systemic inflammation (AST) (*P* < 0.05) in skeletal muscle, while enhancing thermogenesis (UCP1) (*P* < 0.01) in eWAT. GMI treatment promoted myogenesis, enhanced oxygen consumption, and ameliorated STAT3 (*P* < 0.01) through DNAJA3 activation (*P* < 0.05) *in vitro*.

**Conclusions:**

Muscular *Dnaja3* haploinsufficiency dysregulates mitochondrial function and lipid metabolism then leads to sarcopenic obesity. GMI emerges as a therapeutic regimen for sarcopenic obesity treatment through DNAJA3 activation.

## Introduction

Skeletal muscles are the most abundant tissue (40–50% of the total mass) and protein reservoir in the human body; they not only control locomotion but also enact fundamentally for energy expenditure. In addition, skeletal muscles play a crucial role in the homeostasis of glucose, lipids, and proteins, which contributes significantly to overall metabolic health, hence to maintain the high quality of life^1^ ^[S1]^. Although medical advancements have extended human life expectancy, they do not necessarily improve an individual's quality of life or health span because of the human ageing process. In fact, ageing is always associated with increasing incidences of age‐related diseases such as osteoporosis, neurodegenerative disorders and muscle wasting.^2^ Muscle wasting, which refers to the age‐related loss of muscle mass and function or sarcopenia, can result in a 30–50% decrease in skeletal muscle mass and, consequently, reduce muscular force in the elderly population. Muscle wasting or sarcopenia is a hallmark of ageing that contributes to mobility impairment, physical frailty, and increased morbidity in the elderly^3^ ^[S2,S3]^. Further, sarcopenia can be accompanied with obesity due to metabolic change, such as acute and chronic diseases.

Sarcopenic obesity, in the name of concurrence of sarcopenia and obesity, is the co‐existence of excess fat mass and low muscle mass/function.^4^ Fat accumulation causes imbalanced production of adipokines and infiltration of macrophages in adipose tissues^5^; then the pro‐inflammatory cytokines and chemokines can induce systemic and even chronic low‐grade inflammation.^6^ Together, the process of inflammation exerts the development and progression of sarcopenia and impairs insulin sensitivity.^7^ Obesity is caused by increasing production of fatty acids that are deposited in adipose tissues; in addition, the fatty acids can also be distributed to other tissues such as liver and skeletal muscle. Fatty acids can be accumulated in skeletal muscle to form intramuscular adipose tissue (IMAT) and intramyocellular lipids (IMCLs)^8^ ^[S4]^, which leads to lipotoxic effect in skeletal muscle determined by damaged single‐fibre contractility and causes lower muscle strength in elderly co‐relating to decrease insulin sensitivity.^9^ Molecularly and biochemically, the impaired mitochondria β‐oxidation and accumulated reactive oxygen species (ROS) can induce apoptosis or autophagy^10^; together, these can be the potential mechanisms of obesity‐mediated sarcopenia pathogenesis ^[S3]^.^11^

DNAJA3, also known as Tid1 (tumourous imaginal disc 1), is a mammalian mitochondrial DNAJ/HSP40 co‐chaperone protein homologue to the *Drosophila* tumour suppressor protein Tid56.^12^ DNAJA3 contains a conserved J‐domain by which to interact with heat shock protein 70 (HSP70) family members via stimulation of ATPase activity.^13^ Previously, we have demonstrated DNAJA3 plays a vital role in promoting myognesis and maintaining mitochondrial homeostasis in differentiated C2C12 myoblasts. Subsequently, we have shown the importance of DNAJA3 in muscular development and mitochondrial biogenesis by establishing *HSA‐Dnaja3* ^*f/f*^ and *HSA‐Dnaja3* ^*f/+*^ transgenic mice (mice with *Dnaja3* deficiency specifically in skeletal muscle), which show severe muscular dystrophy with reduced motor activity, accompanied with impairment of activity of ATP sensor (p‐AMPK) and mitochondrial biogenesis protein, peroxisome proliferator activated receptor gamma coactivator‐1 alpha (PGC‐1α).^14^ Meanwhile, the *HSA‐Dnaja3* ^*f/f*^ transgenic mice are postnatal lethal around 10 days after the birth because of the severe muscular dystrophy.^14^ Furthermore, Justice et al. have indicated that the genetic variant of human DNAJA3 is highly associated with obesity.^15^

GMI, an immunomodulatory protein cloned from *Ganoderma microsporum*, is found to exhibit anti‐inflammatory effect.^16^ GMI has also been studied comprehensively for the anti‐cancerous treatment through the regulation of immune system ^[S5]^. Hsin et al. have showed that GMI can inhibit tumourigenicity and induce autophagy cell death in non‐small lung cancer cell lines^16^ ^[S5,S6]^. Intriguingly, we recently have demonstrated that GMI can promote myogenic differentiation of C2C12 myoblasts in the manner of DNAJA3 activation.^17^

In this study, we aim to elucidate the physiological defect of muscular *Dnaja3* haploinsufficiency on mitochondrial dysfunction and dysregulated lipid metabolism and subsequently to find out the potential therapeutic regimen, GMI, via activating DNAJA3 to enhance mitochondrial homeostasis to rescue the risk of sarcopenic obesity.

## Methods

### Animals

*Dnaja3* floxed mice were generated according to previous study.^14^ Muscle‐specific *Dnaja3* knockout C57BL/6 mice were generated using the human alpha‐skeletal actin (HSA) promoter Cre‐LoxP system. Mice were given *ab libitum* access to food. The genotyping of the *HSA‐Cre* ^*+/−*^ transgene and the *Dnaja3*‐deficient mice (*HSA‐Dnaja3* ^*f/f*^ or *HSA‐Dnaja3* ^*f/+*^) was identified by polymerase chain reaction (PCR) using genomic DNA isolated from the tail (Supplemental Table)S4. Body weight was measured once a week. The tissues were immediately isolated from euthanized mice, weighted, and stored at −80°C until analysis (Supplemental Methods)S7. All procedures involving mice are approved by the Institutional Animal Care and Use Committee (IACUC No. 1080621n, 1100330n, 1100710, and 1110404r) at National Yang Ming Chiao Tung University (NYCU).

### Fatigue exercise stress test

The procedure of exercise tolerance test was modified from Dougherty et al. ^[S14]^. Briefly, the mice were placed on the treadmill and started running at 12 m/min, subsequently with an incremental speed 2 m/min every 10 min until it reached 28 m/min or the mice reached fatigue. Fatigue condition was defined as the inability to continue running or resting at the starting point for at least 5 s.

### Micro‐computed tomography

Mice were anaesthetised using combination of xylazine (3 mg/kg) and ketamine (20 mg/kg) at 13 months old. Images were visualized by micro‐computed tomography (micro‐CT; MILabs, Utrecht, Netherlands). The mice were imaged using 480 projections. The X‐ray parameters were set at 0.48 mA and 50 kV. Finally, an image voxel was constructed using 80 μm. The images were analysed using 3D‐Slicer and segmented into different body compositions according to tissue density in Hounsfield units (HU). The threshold for whole body fat tissue volume was −350 to −150 HU, and the whole‐body fat free mass volume (lean mass including muscles and organs) was −30 to 200 HU. Micro‐CT estimated tissue weight was calculated by volumetric density of fat mass (0.95 g/cm^3^) and lean mass (1.05 g/cm^3^). After acquiring the volumes in voxels in cm^3^ for fat tissue and fat free tissues, the amount of body fat and lean mass were presented in grams, and then the percentage of fat mass and lean mass was normalized to each body weight.

### *Ganoderma microsporum* immunomodulatory protein treatment

For short‐term treatment, 4‐week‐old male mice were injected intraperitoneally with 1.6 or 3.2 mg/kg *Ganoderma microsporum* immunomodulatory protein (GMI) or saline, as a control group every other day for 1 month, respectively. For long‐term treatment, 3‐month‐old male mice were injected intraperitoneally with 1.6 or 3.2 mg/kg GMI or saline, as a control group every other day for 6 months, respectively.

### Statistical analysis

The statistical analysis was performed by using GraphPad Prism 8 (GraphPad Software, California, USA). Differences among groups were tested by one‐way ANOVA. Comparisons between two groups were performed by the unpaired *t*‐test. *P* values <0.05 was considered significant.

## Results

### Fatigue exercise stress inducing pro‐inflammation, systemic serum inflammation, lipogenesis, and oxidative stress of the young *HSA‐Dnaja3* ^*f/+*^ transgenic mice both *in vivo* and *in vitro*

**Figure 1:**
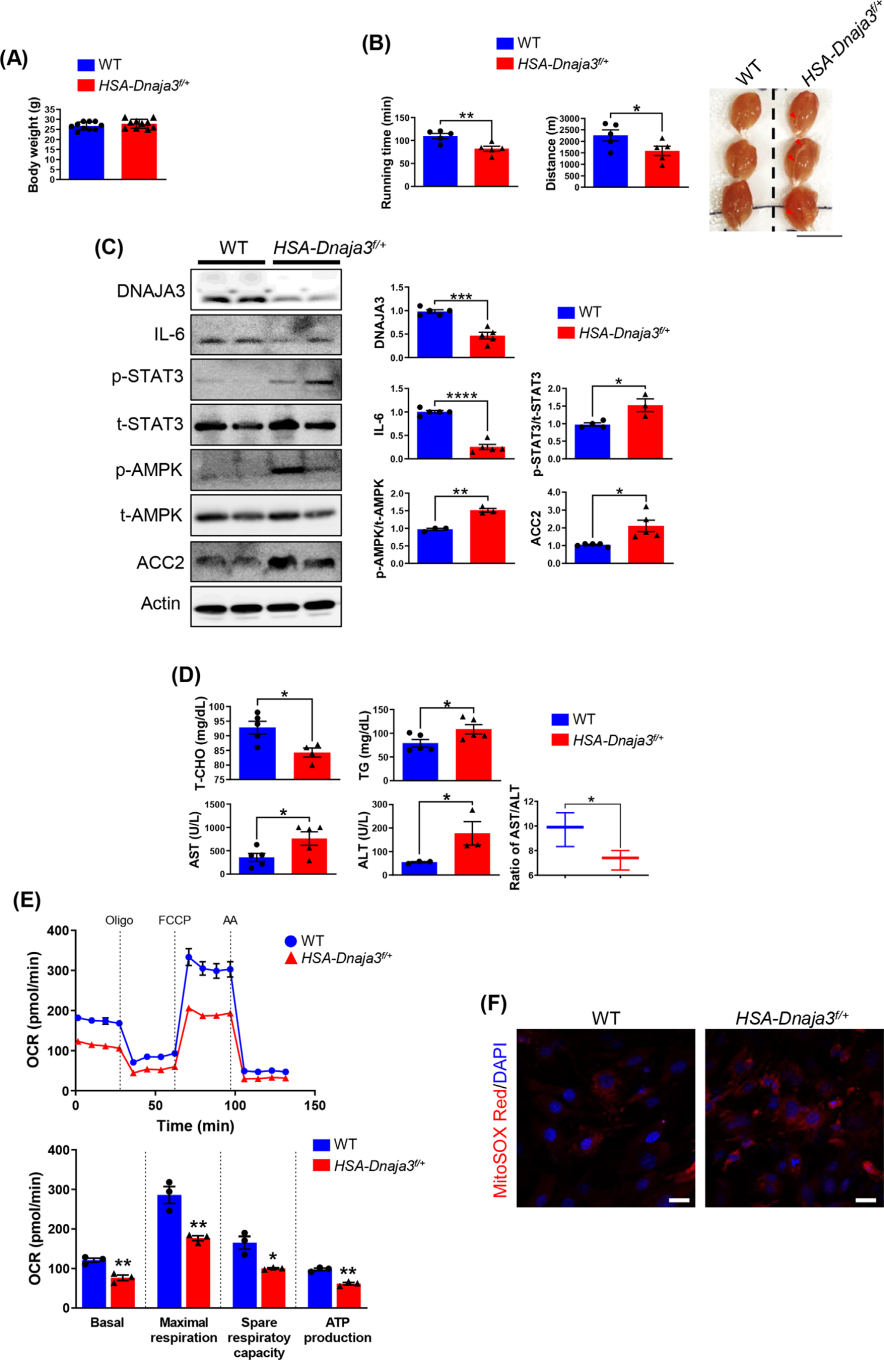
Fatigue exercise stress inducing pro‐inflammation, systemic serum inflammation, lipogenesis and oxidative stress of the young *HSA‐Dnaja3* ^*f/+*^ transgenic mice both *in vivo* and *in vitro*. (A) Body weights of WT mice and *HSA‐Dnaja3* ^*f/+*^ mice were measured at 2‐month‐old (*n* = 10 per group). (B) The running distance and running time of the WT and *HSA‐Dnaja3* ^*f/+*^ mice were recorded (*left* and *middle* panels; *n* = 5 per group). The representative images of gastrocnemius were collected from 2‐month‐old *HSA‐Dnaja3* ^*f/+*^ mice after exercise (*right* panel). The red arrows indicated the sites of haemorrhage (scale bar, 1 cm). (C) After exercise fatigue test, the total protein extracts of gastrocnemius muscle were collected from *HSA‐Dnaja3* ^*f/+*^ and WT mice, respectively, and subject to immunoblot assays. The bar graph data summarized the quantification of the difference between the WT and *HSA‐Dnaja3* ^*f/+*^ mice. Quantification of protein levels was normalized with the amount of actin (*n* = 3–5 per group). (D) The blood serum was collected from WT and *HSA‐Dnaja3* ^*f/+*^ mice after exercise, consequently, the serum levels of triglycerides (TG), total cholesterol (T‐CHO), and the enzymatic activities of alanine aminotransferase (ALT) and aspartate aminotransferase (AST) were examined (*n* = 3–5 per group), respectively. (E) Primary myoblasts were isolated from hindlimbs of 6‐week‐old WT or *HSA‐Dnaja3* ^*f/+*^ mice, respectively. Then, the oxygen consumption rate (OCR) of the induced myoblasts undergoing myogenesis was further analysed by Seahorse XFe24 Extracellular Flux Analyzer (Oligo, oligomycin; FCCP, carbonyl cyanide‐4‐(trifluoromethoxy) phenylhydrazone; AA, antimycin A). Real‐time quadruplicate readings and mitochondrial respiration rates of basal, maximal respiration, spare respiratory capacity, and ATP production were collected (*n* = 3 per group). (F) The confocal representative images of the primary myoblasts, isolated from gastrocnemius muscle of 6‐week‐old WT and *HSA‐Dnaja3* ^*f/+*^ mice after induced differentiation, stained with 5 μM MitoSOX Red, and the nuclei counterstained with DAPI, were collected (scale bar, 20 μm). Statistical analyses were performed by unpaired *t*‐test, mean ± SEM. *P < 0.05, **P < 0.01, ***P < 0.001, ****P < 0.0001.

Our earlier study has demonstrated the importance of DNAJA3 in muscular development and mitochondrial biogenesis through our established *HSA‐Dnaja3* ^*f/f*^ and *HSA‐Dnaja3* ^*f/+*^ transgenic mice (mice with muscular *Dnaja3* null and heterozygosity, respectively). Meanwhile, the *HSA‐Dnaja3* ^*f/f*^ null mice are postnatal lethal around 10 days after the birth that limits the further study.^14^ Herein, instead of using of the *HSA‐Dnaja3* ^*f/f*^ mice, we used the *HSA‐Dnaja3* ^*f/+*^ heterozygous mice to further study the energy homeostasis of mice after exercise stress. First, we did not observe significant difference in body weight change between young WT and *HSA‐Dnaja3* ^*f/+*^ mice (*Figure* 1A). However, the motor coordination abilities (both the running distance and running time) of the *HSA‐ Dnaja3* ^*f/+*^ mice were significantly decreased (*Figure* 1B, left and middle panels). In addition, the gastrocnemius muscles collected from the *HSA‐Dnaja3* ^*f/+*^ mice displayed more reddish and haemorrhages compared with that of the WT mice after exercise (*Figure* 1B, right panel). We further examined the molecular mechanisms involved in muscular homeostasis after exercise. The immunoblotting analyses showed that the amount of pro‐inflammation related marker, IL‐6, was significantly down‐regulated, whereas the level of phosphorylated STAT3 (p‐STAT3) was significantly up‐regulated for the *HSA‐Dnaja3* ^*f/+*^ mice after exercise. Additionally, the amounts of the phosphorylated AMPK and lipogenesis marker, acetyl carboxylase 2 (ACC2), were both significantly up‐regulated (*Figure* 1C). Furthermore, the level of serum total cholesterol (T‐CHO) was significant down‐regulated but the level of serum triglycerides (TG) was significantly increased in the *HSA‐Dnaja3* ^*f/+*^ mice after exercise, indicating the alteration of lipid metabolism. Meanwhile, the levels of serum ALT and AST were both significantly increased, suggesting an induced systemic inflammation in the *HSA‐Dnaja3* ^*f/+*^ mice (*Figure* 1D).

DNAJA3 is a mitochondrial co‐chaperon protein and acts as vital role in maintaining mitochondrial function. Here, we measured the mitochondrial oxygen consumption (OCR) capacity of the isolated primary myoblasts from WT and *HSA‐Dnaja3* ^*f/+*^ mice hindlimbs, after induced myogenesis. We revealed that the *Dnaja3* haploinsufficiency consistently exhibited lower basal, mitochondrial maximal OCR, and spare respiratory capacity in the induced primary myotubes collected from the *HSA‐Dnaja3* ^*f/+*^ mice hindlimbs (*Figure* 1E). Further, we also observed higher mitochondrial ROS accumulation in the *Dnaja3* heterozygous primary myoblasts during myogenesis compared with that of the WT primary myoblasts (*Figure* 1F). Overall, these data indicate that *Dnaja3* haploinsufficiency exerts mitochondrial dysfunction, subsequently decreases motor coordination ability and promotes lipogenesis and systemic inflammation.

### Induced muscular mitochondrial dysfunction atrophying muscular cross‐sectional area and promoting myofibrosis of the young *HSA‐Dnaja3* ^*f/+*^ mice

**Figure 2:**
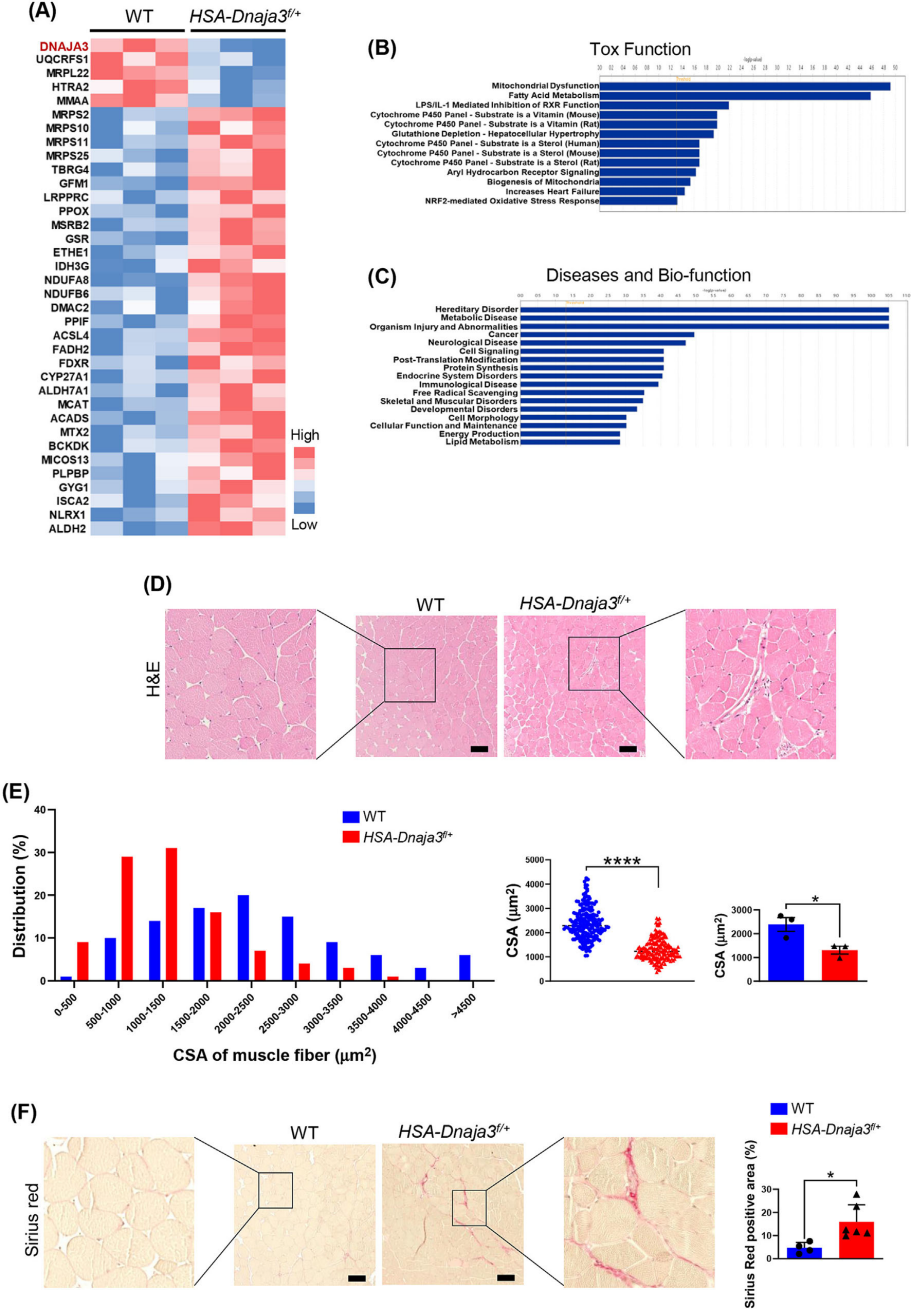
Induced muscular mitochondrial dysfunction atrophying muscular cross‐sectional area and promoting myofibrosis of the young *HSA‐Dnaja3* ^*f/+*^ mice. Mitochondrial fractionated proteins of gastrocnemius muscle were collected from 2‐month‐old WT and *HSA‐Dnaja3* ^*f/+*^ mice (*n* = 3 per group) and subjected to LC–MS/MS, respectively. (A) The differential expression profiles of proteins from mitochondrial‐enriched fraction of WT and *HSA‐Dnaja3* ^*f/+*^ mice were presented as heatmaps (*blue*, down‐regulated; *red*, up‐regulated). (B, C) The charts presented the top significant associated biological processes, tox function, and diseases with bio‐function analysed by ingenuity pathways analysis (IPA). (D–F) Gastrocnemius muscles were collected at 2‐month‐old WT or *HSA‐Dnaja3* ^*f/+*^ mice, respectively, after exercise fatigue test. (D) The representative histological pictures were collected from the haematoxylin and eosin (H&E)‐stained gastrocnemius muscle sections (scale bar, 100 μm). (E) The quantification of cross‐sectional area (*left*), the relative frequency distribution (%) of muscle myofibers size (*middle*) and the mean of myofibers size (*right*) (*n* = 3 per group) of the gastrocnemius muscle were quantified. (*F*) The characteristic images were collected from the gastrocnemius muscle sections stained with Sirius Red (scale bar, 100 μm), and the Sirius Red positive areas were quantified (WT *n* = 4, *HSA‐Dnaja3* ^*f/+*^ *n* = 6). Statistical analyses were performed by unpaired *t*‐test, mean ± SEM. ****P < 0.0001.

**Figure 3:**
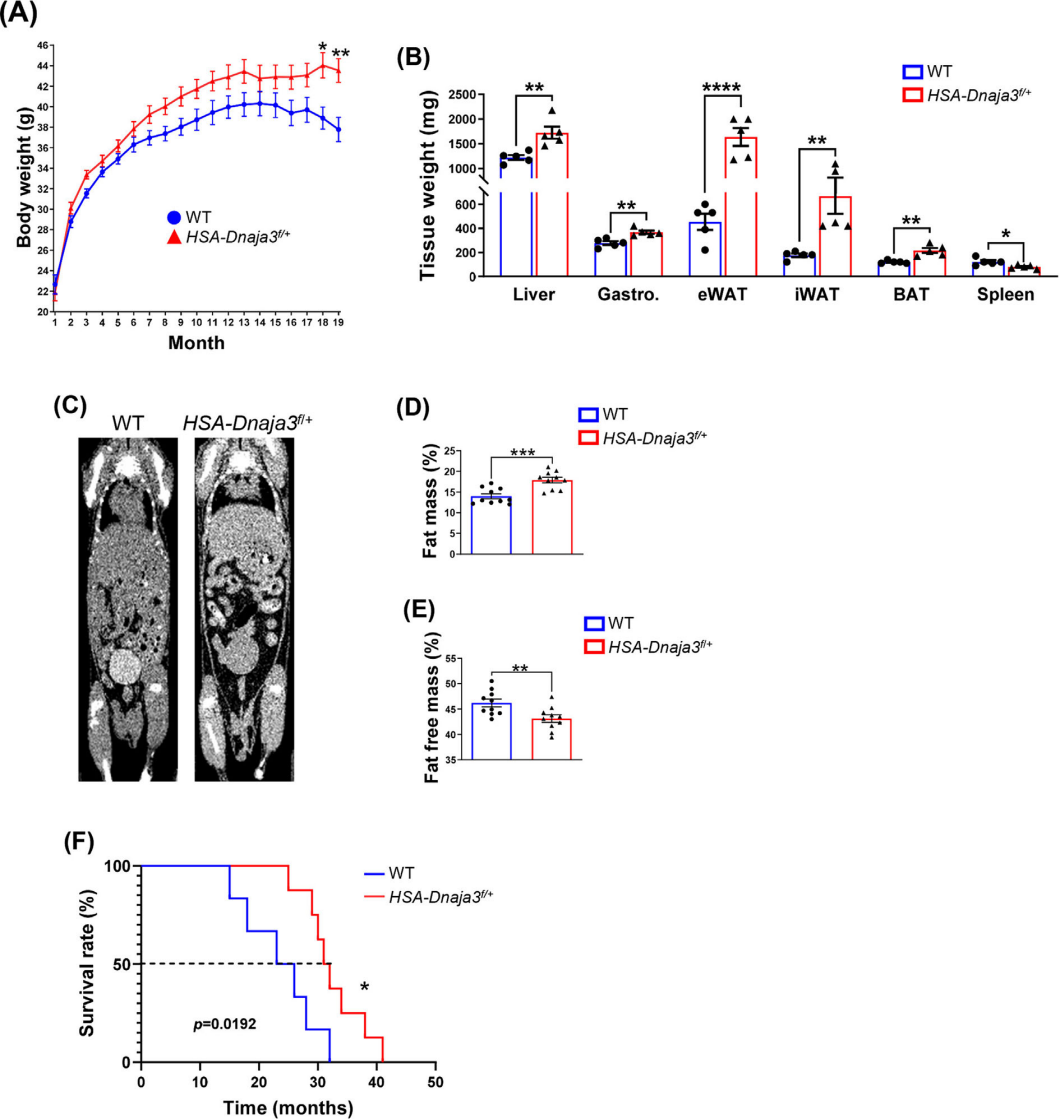
Muscular *Dnaja3* heterozygosity promoting fat accumulation and inducing the potency of sarcopenic obesity during ageing. (A) The body weight curves of WT and *HSA‐Dnaja3* ^*f/+*^ mice were monthly recorded (*n* = 15 per group). (B) The 13‐month‐old WT and *HSA‐Dnaja3* ^*f/+*^ mice were sacrificed; the bar graph data summarized the tissues' weight of liver, gastrocnemius muscle, adipose tissues (eWAT, epididymal white adipose tissue; iWAT, inguinal white adipose tissue; BAT, brown adipose tissue) and spleen (*n* = 5 per group). (C) The representative images of 3D whole‐body of the 13‐month‐old WT and *HSA‐Dnaja3* ^*f/+*^ mice were acquired by micro‐CT scan *in vivo*. The fat mass (D) and fat‐free mass (E) (including muscles and organs, bone excluded) of the WT and *HSA‐Dnaja3* ^*f/+*^ mice were analysed by 3D‐slicer (*n* = 10 per group). (F) The overall survival rates of the WT and *HSA‐Dnaja3* ^*f/+*^ mice were recorded (*n* = 10 per group). The dotted horizontal line indicated the median survival time for WT (24.5 months) and *HSA‐Dnaja3* ^*f/+*^ mice (31.5 months). Statistical analyses were performed by unpaired *t*‐test, mean ± SEM. *P < 0.05, **P < 0.01, ***P < 0.001, ****P < 0.0001.

Mitochondria are vital organelles to regulate the metabolism state and balance the reactive oxygen species (ROS) level in skeletal muscle,^18^ and DNAJA3, acting as a mitochondrial co‐chaperon protein, plays a crucial role in maintaining mitochondrial DNA,^19^ membrane potential,^20^ and cristae structure^21^ and in balancing both the intracellular and mitochondrial ROS levels.^22^ To determine whether *Dnaja3* heterozygosity impaired mitochondrial homeostasis, firstly, we isolated the mitochondria from the gastrocnemius muscles of 2‐month‐old WT and *HSA‐Dnaja3* ^*f/+*^ mice after exercise fatigue test, respectively. Consequently, the immunoblotting assays were examined to show that the protein level of DNAJA3 was significantly reduced; however, the protein levels of VDAC and OXPHOS complexes were not significantly different in the mitochondria isolated from muscle tissues of *HSA‐Dnaja3* ^*f/+*^ mice (*Figure* S1). Further, the isolated muscular mitochondria from the WT and *HSA‐Dnaja3* ^*f/+*^ mice were subject to LC–MS/MS to detect the differential mitochondrial proteomes. In total, 1434 proteins were identified by LC–MS/MS. As shown in Figure 3A, of the 36 significantly differential mitochondrial proteins from systematic analyses, including DNAJA3, its protein level was reduced; we also observed that in particular for the mitochondrial respiratory proteins, UQCRFS1, its protein level was decreased whereas for NDUFA8 and NDUFB6, their protein levels were increased in the *HSA‐Dnaja3* ^*f/+*^ mice. For mitochondrial transcription and translation proteins, we found that the protein levels of MRPL22 was decreased, but the protein levels of MRPS2, MRPS10, MRPS11, and MRPS25 were increased in *Dnaja3* heterozygous mice. Meanwhile, the amounts of proteins involved in fatty acid and cholesterol metabolism, such as ACSL4, ACADS, MCAT, and so on, were up‐regulated in the mitochondrial proteome of *HSA‐Dnaja3* ^*f/+*^ mice (*Figure* 2A). Additionally, the ingenuity pathway analysis (IPA) showed the gene ontology (GO) with significant 13 top associated biological processes including mitochondrial dysfunction, fatty acid metabolism, and oxidative stress response (*Figure* 2B); the differential mitochondrial proteome analyses indicated that *Dnaja3* heterozygosity could affect muscular disorders and lipid metabolism (*Figure* 2C).

To further investigate the histopathology of skeletal muscular *Dnaja3* deficiency, we collected the gastrocnemius muscles from the 2‐month‐old WT or HSA‐*Dnaja3* ^f/+^ mice after exercise fatigue test and performed H&E staining. We observed that more nuclei in the muscle tissues of *HSA‐Dnaja3* ^*f/+*^ mice, suggesting the undergoing inflammation along with the infiltrating lymphocytes (*Figure* 2D). In addition, the cross‐sectional area (CSA) and muscle fibre size in muscular of the gastrocnemius muscle *Dnaja3* heterozygosity mice were smaller (*Figure* 2E). Further, the Sirius red stain was administrated to show severe myofibrosis in the muscle tissues of *HSA‐Dnaja3* ^*f/+*^ mice in comparison with that of the WT mice (*Figure* 2F). Together, these findings suggest that DNAJA3 plays a vital role in regulating mitochondrial proteostasis and ribosomes, meanwhile induces muscle atrophy and myofibrosis.

### Muscular *Dnaja3* heterozygosity promoting fat accumulation and inducing the potency of sarcopenic obesity during ageing

We observed that the *Dnaja3* heterozygosity impaired the mitochondrial homeostasis, which prompts us to further monitor the pathophysiological effects in mediating the sarcopenic obesity in the *HSA‐Dnaja3* ^*f/+*^ mice during ageing. Firstly, we observed the body weights of the *HSA‐Dnaja3* ^*f/+*^ mice were significantly increased at 18 months (*Figure* 3A). Additionally, the weighed amounts of epididymal white adipose tissues (eWAT) and inguinal white adipose tissues (iWAT) excised from the *HSA‐Dnaja3* ^*f/+*^ mice were significantly larger than those from the WT mice; meanwhile, the liver, gastrocnemius muscle, and brown adipose tissues (BAT) weight were also increased in the *HSA‐Dnaja3* ^*f/+*^ mice. In addition, we observed that the spleen weight was significantly smaller in the *HSA‐Dnaja3* ^*f/+*^ mice (*Figure* 3B). To further evaluate whether *Dnaja3* heterozygosity affects fat accumulation in the body, we chose a non‐invasive methodology by using micro‐CT to validate the alterations of body composition (i.e., fat and fat free). Of note, we observed that the whole body fat mass was significantly increased, whereas the whole body fat free mass was significantly decreased in the *HSA‐Dnaja3* ^*f/+*^ mice in comparison with the WT mice (*Figure* 3C–E). Additionally, we observed more intramuscular lipid droplet accumulation in the primary cells isolated from the hindlimbs of 6‐week‐old *HSA‐Dnaja3* ^*f/+*^ mice by using oil‐red‐O staining (*Figure* S2A,B). Surprisingly, the *HSA‐Dnaja3* ^*f/+*^ mice displayed a prolonged lifespan, having a median of 31.5 months of 50% survival time, in comparison with the WT mice (24.5 months) (*Figure* 3F). Altogether, these results indicate that muscular *Dnaja3* heterozygosity promotes the risk of sarcopenic obesity during ageing.

### Promoting the potency of sarcopenic obesity and increasing the risk of insulin resistance in the aged *HSA‐Dnaja3* ^*f/+*^ mice

**Figure 4:**
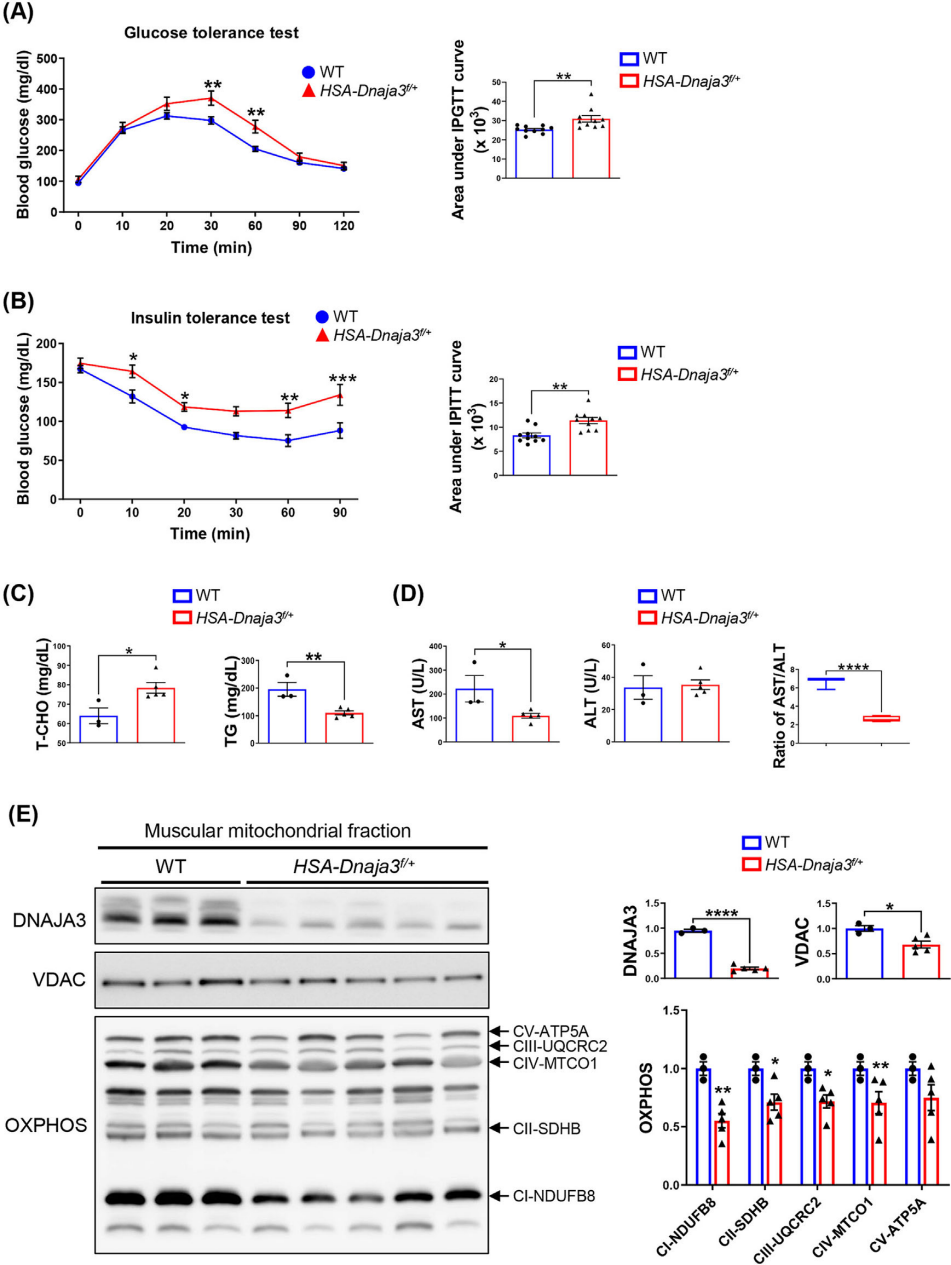
Promoting the potency of sarcopenic obesity and increasing the risk of insulin resistance in the aged *HSA‐Dnaja3* ^*f/*+^ mice. The glucose tolerance (A) and insulin tolerance (B) tests were examined on 13 or 14‐month‐old WT and *HSA‐Dnaja3* ^*f/+*^ mice, respectively (*n* = 10 per group). The blood serum was collected from 13‐month‐old WT (*n* = 3) and *HSA‐Dnaja3* ^*f/+*^ mice (*n* = 5); consequently, the serum levels of T‐CHO and TG (C), and the enzymatic activities of ALT and AST (D) were examined, respectively. (E) Mitochondrial fractionated proteins of gastrocnemius muscle were collected from 13‐month‐old WT and *HSA‐Dnaja3* ^*f/+*^ mice, respectively. The immunoblotting assays against the mitochondrial proteins including DNAJA3, voltage‐dependent anion channel (VDAC), and mitochondrial oxidative phosphorylation (OXPHOS) complexes were analysed; the bar graph data summarized the quantification of the difference between the aged WT and *HSA‐Dnaja3* ^*f/+*^ mice (WT *n* = 3, *HSA‐Dnaja3* ^*f/+*^ *n* = 5). Statistical analyses were performed by unpaired *t*‐test, mean ± SEM. *P < 0.05, **P < 0.01, ****P < 0.0001.

Sarcopenic obesity can increase the risk of metabolic disorders, such as insulin resistance.^23^ To determine whether muscular *Dnaja3* heterozygosity would deregulate systemic glucose metabolism and cause hyperglycaemia, we assessed glucose homeostasis with glucose tolerance and insulin tolerance tests, respectively. As shown in Figure 4A,B, compared with the WT mice, we observed the less glucose tolerance and impaired sensitivity to insulin tolerance in the *HSA‐Dnaja3* ^*f/+*^ mice. In addition, we found that the level of serum total cholesterol (T‐CHO) was significantly increased whereas the level of serum triglycerides (TG) was significantly decreased in the *HSA‐Dnaja3* ^*f/+*^ mice (*Figure* 4C). Further, we detected that the level of serum ALT was no difference; however, the level of serum AST was significantly declined in *HSA‐Dnaja3* ^*f/+*^ mice (*Figure* 4D). Previous study has indicated that occurrence of obesity and type 2 diabetes mellitus (T2MD) tends to decrease the ratio of AST/ALT.^24^ Of note, the ratio of serum AST/ALT level was significantly lower in the muscular *Dnaja3* heterozygosity mice in comparison with that of the WT mice (*Figure* 4D). Taken together, muscular *Dnaja3* heterozygosity can induce sarcopenic obesity, impede the blood glucose homeostasis, and raise the risk of diabetes mellitus.

### Disrupting mitochondrial proteostasis in skeletal muscles of the aged *HSA‐Dnaja3* ^*f/+*^ mice

To further elucidate whether muscular *Dnaja3* heterozygosity would dysregulate mitochondrial proteostasis in aged muscles, we isolated mitochondria from gastrocnemius muscles of the 13‐month‐old WT and *HSA‐Dnaja3* ^*f/+*^ mice, respectively. As expected, the protein level of DNAJA3 was significantly reduced; meanwhile, the protein levels of VDAC and mitochondrial OXPHOS were also significantly decreased in the aged *HSA‐Dnaja3* ^*f/+*^ mice in comparison with those of the WT mice (*Figure* 4E). Together, the results suggest that muscular *Dnaja3* heterozygosity dysregulates mitochondrial proteostasis during ageing.

### Early initiation of short‐term GMI treatment ameliorating exercise capacity and inflammation by restoring DNAJA3 expression *in vivo*

**Figure 5:**
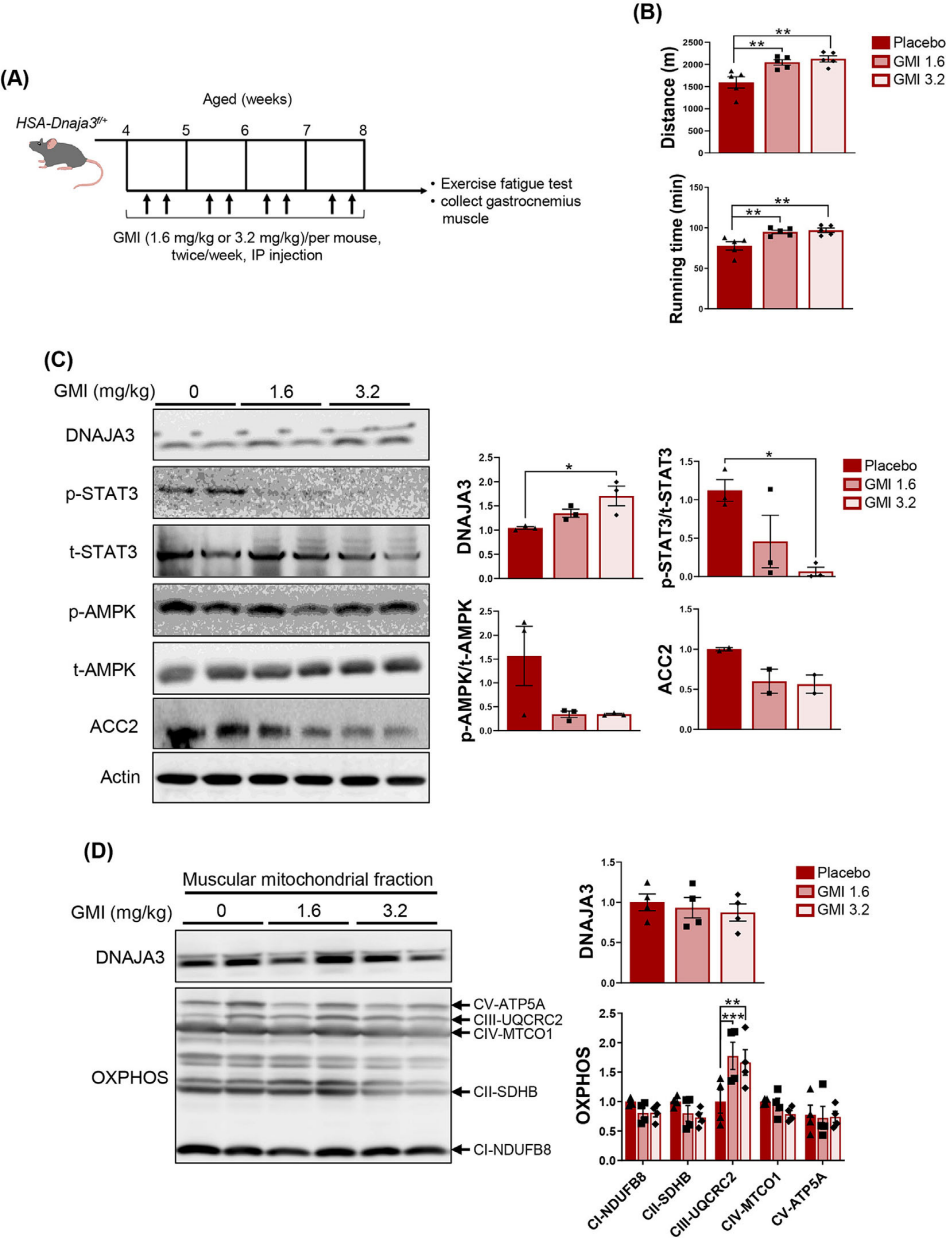
Early initiation of short‐term GMI treatment ameliorating exercise capacity and inflammation by restoring DNAJA3 expression of the *HSA‐Dnaja3* ^*f/+*^ mice *in vivo.* (A) The scheme depicting GMI treatment on *HSA‐Dnaja3* ^*f/+*^ mice *in vivo*. The 4‐week‐old *HSA‐Dnaja3* ^*f/+*^ mice were subject to intraperitoneal injection with placebo, 1.6 or 3.2 mg/kg of GMI treatment, twice a week consecutively for 1 month; then the locomotion abilities were measured and the gastrocnemius muscle were collected for further examination. (B) The abilities of running distance and running time of the *HSA‐Dnaja3* ^*f/+*^ mice treated with placebo or GMI‐treated groups were recorded (*n* = 5 per group). (C) The total protein extracts of gastrocnemius muscle were collected from 2‐month‐old *HSA‐Dnaja3* ^*f/+*^ mice treated with placebo or GMI‐treated groups after exercise fatigue test. The protein levels of DNAJA3, STAT3, AMPK, and ACC2 were analysed by immunoblot; the bar graph data summarized the quantification of the placebo or GMI‐treated groups (*n* = 2–3 per group). (D) The crude mitochondrial fractionated proteins of gastrocnemius muscle were isolated from *HSA‐Dnaja3* ^*f/+*^ mice treated with placebo or GMI after exercise fatigue test. The immunoblotting assays against the mitochondrial proteins including DNAJA3, voltage‐dependent anion channel (VDAC), and mitochondrial oxidative phosphorylation (OXPHOS) complexes were analysed; the bar graph data summarized the quantification of the placebo or GMI‐treated groups (*n* = 4 per group). Statistical analyses were performed by one‐way ANOVA, mean ± SEM. *P < 0.05, **P < 0.01, ***P < 0.001.

Previously, we find that GMI exerts the anti‐inflammatory effect and promote myogenesis, accompanied with up‐regulating the amount of DNAJA3 in C2C12 cells.^17^ To determine the beneficial effect of GMI treatment on *HSA‐Dnaja3* ^*f/+*^ mice *in vivo*, the mice were treated with 1.6 or 3.2 mg/kg of GMI and subsequently examined with exercise fatigue test, respectively (*Figure* 5A). Herein, we observed that GMI treatment significantly improved the exercise performances (*Figure* 5B). Furthermore, as showed in *Figure* 5C, after fatigue exercise, the amount of DNAJA3 was enhanced in the *HSA‐Dnaja3* ^*f/+*^ mice treated with GMI in a dose‐dependent manner. Additionally, the protein level of inflammation related marker, phospho‐STAT3, was down‐regulated in a dose‐dependent manner (*Figure* 5C). Additionally, the amount of phospho‐AMPK was also decreased in the *HSA‐Dnaja3* ^*f/+*^ mice treated with GMI. Meanwhile, lipogenesis marker, ACC2, was down‐regulated in the *HSA‐Dnaja3* ^*f/+*^ mice treated with GMI, suggesting that GMI treatment alleviates the lipid droplet in the skeletal muscle (*Figure* 5C).

Consequently, we isolated the mitochondria from gastrocnemius muscles of *the HSA‐Dnaja3* ^*f/+*^ mice untreated or treated with GMI. Immunoblot assay was used to show that the protein levels of DNAJA3 and VDAC were no difference after GMI treatment. Nevertheless, the amount of mitochondrial respiratory complex III (subunit UQCRC2) was significantly increased in the *HSA‐Dnaja3* ^*f/+*^ mice treated with GMI (*Figure* 5D). Collectively, the GMI treatment not only enhances muscle locomotion but also ameliorates inflammation response and lipogenesis via DNAJA3 activation.

### Long‐term GMI treatment alleviating the pathophysiological symptoms of sarcopenic obesity and inducing browning of WAT in muscular *Dnaja3* heterozygous mice

**Figure 6:**
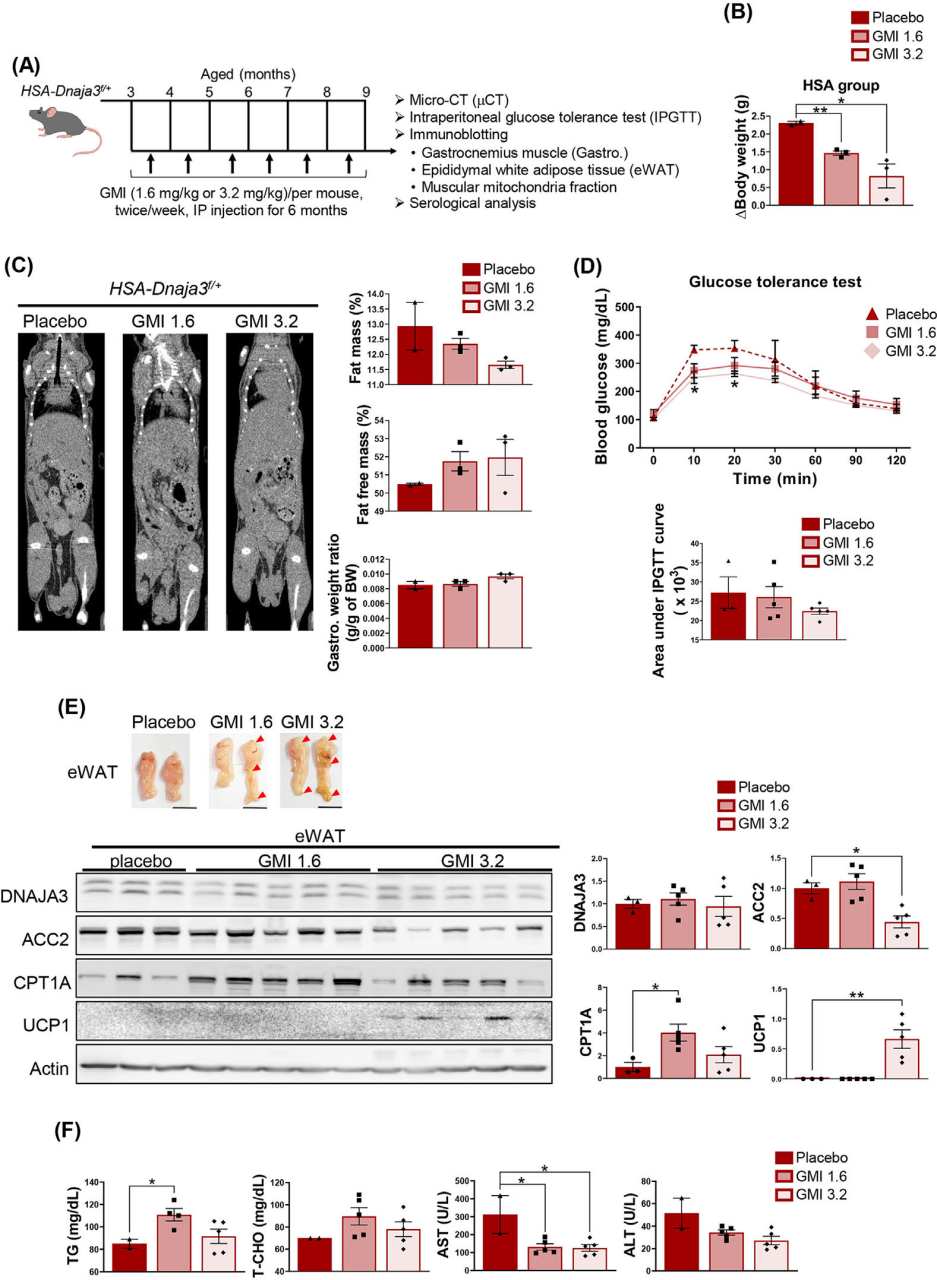
Long‐term GMI treatment alleviating the pathophysiological symptoms of sarcopenic obesity and inducing browning of WAT in muscular *Dnaja3* heterozygous mice. The *HSA‐Dnaja3* ^*f/+*^ mice at 3‐month‐old were intraperitoneally injected with placebo, 1.6 or 3.2 mg/kg of GMI twice a week consecutively for 6 months, respectively. (A) The body weights of *HSA‐Dnaja3* ^*f/+*^ mice after GMI treatment were recorded, respectively (placebo (*n* = 2) or GMI‐treated groups (*n* = 3 per group)). (B) The representative images of 3D whole‐body of the *HSA‐Dnaja3* ^*f/+*^ mice treated with placebo (*n* = 2) or GMI (*n* = 3 per group) at 9‐month‐old were acquired by micro‐CT scan *in vivo* (*left* panel); and the fat mass and fat‐free mass (including muscles and organs, bone excluded) (*right* panel) were measured, respectively. (C) The glucose tolerance tests were measured on *HSA‐Dnaja3* ^*f/+*^ mice treated with placebo or GMI (*n* = 2 to 3 per group). (D) the representative images showed the eWAT tissues excised from *HSA‐Dnaja3* ^*f/+*^ mice treated with placebo or GMI, respectively; the red arrows indicated the sites of browning (scale bar, 1 cm). (E) The total protein extracts of the excised eWAT tissues were subject to immunoblot assays with antibodies against DNAJA3, ACC2, CPT1A, and UCP1 (*lower* panel); the bar graph summarized the quantification of the placebo or GMI‐treated groups. (F) the blood serum was collected from the *HSA‐Dnaja3* ^*f/+*^ mice treated with placebo (*n* = 3), or GMI (*n* = 5 per group); consequently, the enzymatic activities of ALT and AST were examined. Statistical analyses were performed by one‐way ANOVA, mean ± SEM. *P < 0.05, **P < 0.01, ****P < 0.0001.

We observed that the body weight of the *HSA‐Dnaja3^f/+^* mice was increased compared to that of WT mice since 3‐month‐old (*Figure* 3A). Therefore, to further validate whether GMI treatment could alleviate the pathophysiological symptoms of sarcopenic obesity in muscular *Dnaja3* heterozygosity mice *in vivo*, the *HSA‐Dnaja3* ^*f/+*^ mice were treated with 1.6 or 3.2 mg/kg of GMI at the age of 3 months for 6 months (*Figure* 6A). First, we observed the body weight gain of *HSA‐Dnaja3* ^*f/+*^ mice treated with GMI was significantly decreased at high‐dose GMI condition (*Figure* 6B). Moreover, the micro‐CT analysis revealed the whole‐body fat mass was decreased, whereas the whole‐body fat free mass and gastrocnemius muscles were increased in the *HSA‐Dnaja3* ^*f/+*^ mice treated with GMI in a dose‐dependent manner (*Figure* 6C). In addition, we observed the amelioration of glucose tolerance in the *HSA‐Dnaja3* ^*f/+*^ mice with high‐dose GMI treatment (*Figure* 6D), suggesting that GMI treatment enhances the ability of glucose homeostasis.

Meanwhile, we isolated the epididymal white adipose tissues (eWAT) from the *HSA‐Dnaja3* ^*f/+*^ mice treated with or without GMI. Herein, we observed that multiple sites of eWAT were became beige‐like adipocytes after GMI treatment (*Figure* 6E). The immunoblot assay was used to show that the protein levels of DNAJA3 was no significantly difference among the placebo and GMI treated mice, whereas the amount of ACC2 was significantly down‐regulated at high‐dose of GMI treatment in *HSA‐Dnaja3* ^*f/+*^ mice. Moreover, the level of fatty acid oxidation related marker, CPT1A, was significantly increased after treated with GMI. Of note, the amount of thermogenesis marker, UCP1, was significantly up‐regulated at high‐dose of GMI treatment (*Figure* 6E). However, the muscular mitochondria isolated from gastrocnemius muscle of *HSA‐Dnaja3* ^*f/+*^ mice treated with placebo or GMI were no difference (*Figure* S3). Meanwhile, the level of serum TG was significantly increased at low‐dose of GMI treatment in the *HSA‐Dnaja3* ^*f/+*^ mice, whereas the level of serum T‐CHO was no significantly difference among the three groups of mice (*Figure* 6F).Furthermore, the levels of both serum AST and ALT were decreased in the *HSA‐Dnaja3* ^*f/+*^ mice treated with GMI (*Figure* 6F). Taken together, we summarize that long‐term GMI treatment ameliorates muscular *Dnaja3* heterozygosity induced imbalanced muscle homeostasis, fat accumulation, and systemic inflammation and consequently alleviates the pathophysiological symptoms of sarcopenic obesity.

### GMI treatment restoring the myogenesis and mitochondrial respiration ability in *Dnaja3* heterozygous primary myoblasts

**Figure 7:**
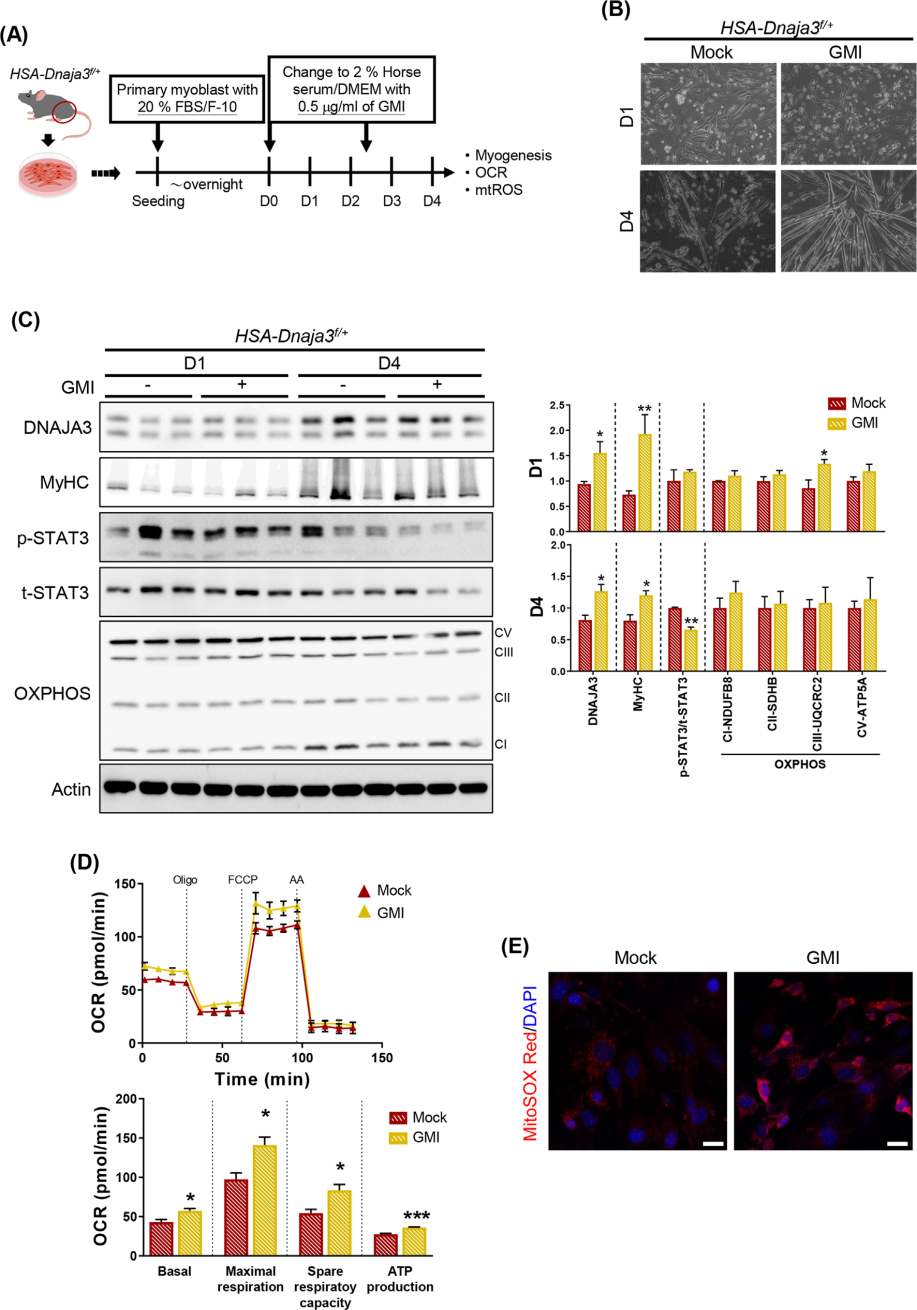
GMI treatment restoring the myogenesis and mitochondrial respiration ability in *Dnaja3* heterozygous primary myoblasts. (A) The scheme depicting the GMI treatment on primary myoblasts isolated from the hindlimbs of 6‐week‐old *HSA‐Dnaja3* ^*f/+*^ undergoing induced myogenesis *in vitro*. (B) The representative phase contrast images of the isolated primary myoblasts, treated with or without GMI upon induced myogenesis at different time intervals (day 1 and 4, respectively), were recorded. (C) The crude protein extracts were collected from primary myoblasts treated with or without GMI after induced myogenesis at day 1 and day 4, respectively, and examined by immunoblot assays with antibodies against DNAJA3, MyHC, p‐STAT3, t‐STAT3, and OXPHOS; the bar graph data summarized the quantification of the differentiated primary myoblasts treated with or without GMI (mock *n* = 3, GMI *n* = 3–4). (D) Then, the oxygen consumption rate (OCR) of the isolated primary myoblasts treated with or without GMI undergoing myogenesis (day 4) was further analysed by Seahorse XFe24 Extracellular Flux Analyzer (*left* panel) (Oligo, oligomycin; FCCP, carbonyl cyanide‐4‐(trifluoromethoxy) phenylhydrazone; AA, antimycin A). The real‐time quadruplicate readings and mitochondrial respiration rates of basal, maximal respiration, spare respiratory capacity and ATP production were collected (*right* panel) (mock *n* = 3, GMI *n* = 4). (E) The confocal representative images of the isolated primary myoblasts (day 4), treated with or without GMI, stained with 5 μM MitoSOX Red, and the nuclei counterstained with DAPI, were collected (scale bar, 20 μm). Statistical analyses were performed by unpaired *t*‐test, mean ± SEM. *P < 0.05, **P < 0.01, ***P < 0.001.

To further evaluated the restoration of GMI in skeletal muscle myogenesis through DNAJA3 activation, we isolated the primary myoblasts from hindlimbs of the *HSA‐Dnaja3* ^*f/+*^ mice, then treated with 0.5 μg/mL GMI during the induced myogenesis *in vitro* (*Figure* 7A). Phenotypically, we observed that the GMI treatment to the *HSA‐Dnaja3* ^*f/+*^ primary myoblasts not only improved myogenic differentiation but also prevented the differentiated myotubes from cell death at day 4 (*Figure* 7B). Meanwhile, the GMI treatment up‐regulated the protein level of mitochondrial protein DNAJA3; in addition, the amount of myogenesis specific marker, MyHC, was also up‐regulated both at day 1 and day 4 during myogenesis (*Figure* 7C). The ratio of phospho‐STAT3/tatol‐STAT3 was significantly reduced in the GMI treated *Dnaja3* heterozygous primary myoblasts at day 4 during myogenesis (*Figure* 7C). Moreover, we found that the GMI treatment up‐regulated the protein level of mitochondrial respiratory complexes (*Figure* 7C), subsequently ameliorating the basal, mitochondrial maximal OCR, and spare respiratory capacity and increasing ATP production of the differentiated *HSA‐Dnaja3* ^*f/+*^ primary myoblasts (*Figure* 7D). However, we observed higher mitochondrial ROS accumulation after GMI treatment in *Dnaja3* heterozygous primary myoblasts during myogenesis (*Figure* 7E). For the phenotypic effect on the WT primary myoblasts under GMI treatment, we, indeed, observed the amelioration of the ability of myogenesis and the up‐regulation of DNAJA3 protein level but not the enhanced mitochondrial respiration (*Figure* S4A–C); subsequently, the mitochondrial ROS level in WT primary myoblasts was higher after treating with GMI (*Figure* S4D). Overall, these data suggest that GMI treatment could restore the defective myogenesis and mitochondrial respiration via DNAJA3 activation but limitedly enhance the mitochondrial function of the WT primary myoblasts during myogenesis.

## Discussion

**Figure 8:**
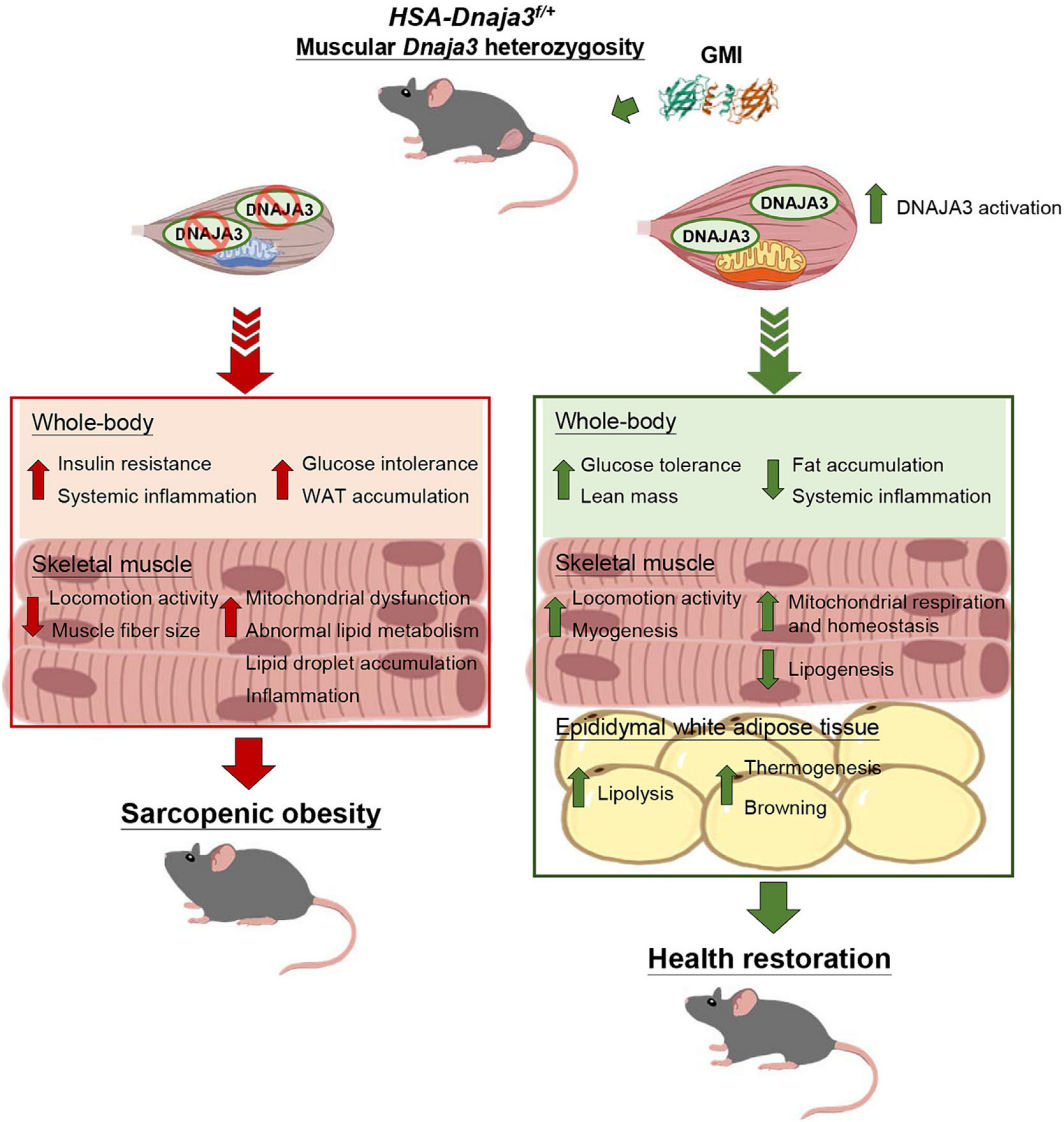
Graphic summary of muscular *Dnaja3* heterozygosity inducing sarcopenic obesity and restoring the pathophysiological symptoms by GMI treatment. GMI structure was adapted from RCSB Protein Data Bank (RCSB PDB, https://www.rcsb.org/structure/3KCW).

Mitochondrial dysfunction is proposed to be the main pathogenesis of sarcopenic obesity and ageing, including reduction of mitochondrial mass, respiratory function, and quality control and exacerbation of oxidative stress and inflammation.^25^ DNAJA3 acts as a mitochondrial co‐chaperon protein, interacting with Hsp70 that is an important complex to maintain mitochondrial cristae structure.^21^ In addition, DNAJA3 has been reported to play a vital role in sustaining a distribution of mitochondrial membrane potential and the integrity of mtDNA in cancer cells.^20^ However, there are few reports on the association with mitochondria homeostasis maintaining mediated by DNAJA3 in skeletal muscle. Previously, we have demonstrated *Dnaja3* knockdown could abrogate mitochondrial membrane potential and reduce intracellular ATP production in C2C12 myoblast cells.^14^ Meanwhile, the myogenesis ability of C2C12 cells can be enhanced by GMI treatment via DNAJA3 activation.^17^ Herein, we demonstrated that muscular *Dnaja3* heterozygosity induced mitochondrial dysfunction, subsequently reduced locomotion activity and muscular cross‐sectional area. Moreover, muscular *Dnaja3* heterozygosity induced metabolic disorders to increase the risk of pathogenesis of sarcopenic obesity. Therefore, GMI could ameliorate and restore the pathological symptoms via DNAJA3 activation both *in vitro* and *in vivo* (*Figure* 8).

The dysregulation of muscular homeostasis and the ageing process is associated with high level of ROS, which impairs myogenesis and muscle regeneration.^26^ Mitochondria are the major organelle to produce ROS.^26^ Our mitochondrial proteome analyses confirmed muscular *Dnaja3* haploinsufficiency, along with down‐regulating the expression complex III related protein, impairing mitochondrial dysfunction, and causing oxidative stress. Subsequently, we observed the high level of mitochondrial ROS in the differentiated primary myoblasts of *Dnaja3* heterozygosity. However, we found that GMI treatment could increase mitochondrial ROS level in *Dnaja3* heterozygosity primary myoblasts during myogenesis (*Figure* 7E). Mitochondrial complexes I and III of electron transport chain (ETC) are the dominant sites to produce ROS,^27^ and mtROS levels are increased during early myogenic differentiation.^28^ These findings suggested that GMI ameliorated mitochondrial respiration through DNAJA3 activation, associating with the up‐regulation of complex III (UQCRC2) protein expression.

Sarcopenia obesity, in the name of coexistence of sarcopenia and obesity, is characterized with decreased muscle mass and increased fat accumulation; together, they lead to defective muscle strength.^29^ Additionally, the inflammatory cytokines secreted from adipose tissues can contribute muscle insulin resistance and impair muscular function.^30^ Conversely, the loss of muscle mass in sarcopenia can lead to a reduction in overall energy expenditure, potentially contributing to the development or exacerbation of obesity.^4^ Our present data showed that muscular *Dnaja3* heterozygosity diminished motor coordination and increased systemic inflammation; meanwhile, the whole‐body of fat mass was increased, and the fat‐free mass was decreased by using micro‐CT (*Figure* 3C). Furthermore, we demonstrated the escalated risk of hyperglycaemia and insulin resistance in aged *Dnaja3* heterozygosity mice (*Figure* 4A,B). Moreover, previous study has validated that *DNAJA3* mutation is highly associated with waist‐to‐hip ratio adjusted for body mass index (WHRadjBMI) indicating *DNAJA3* polymorphism may be highly related to obesity.^15^ Taken together, DNAJA3 can act as a key regulator to mediate the process of sarcopenic obesity during ageing.

Previous study has indicated that sarcopenic obesity may accelerate the risk of mortality.^31^ However, in cancer cachexia or cardiovascular disease (CVD) patients, being accompanied with sarcopenic obesity exhibit better survival rate compared with those who are not accompanied with sarcopenic obesity.^32^, ^33^ Moreover, mitochondrial electron transport chain dysfunction can lead to extend longevity in various species^34^ ^[S7, S8]^. This paradox may indicate the reason why muscular *Dnaja3* heterozygosity, impairing mitochondrial function, subsequently leading to sarcopenic obesity and extended survival (*Figure* 3F).

Acetyl‐CoA carboxylase 2 (ACC2), localized at the mitochondrial surface, is a main regulatory enzyme mediating mitochondrial fatty acid oxidation in skeletal muscle ^[S9]^. Previous study has validated that muscular *ACC2* expression is increased in the situation of obesity, and insulin resistance, oppositely, diminished its expression with weight loss ^[S10]^. Additionally, *ACC2* knockout mice reveal a notable resistance to diet‐induced obesity and diabetes mellitus via the elevation of fatty acid oxidation and energy expenditure ^[S11]^. In our study, we observed the increased protein level of ACC2 in young *Dnaja3* heterozygous mice, whereas *Dnaja3* haploinsufficiency caused fat accumulation to lead to insulin resistance at aged mice. However, the mechanisms of how DNAJA3 mediates ACC2 remain unclear.

Skeletal muscle, liver, and adipose tissues are the major organs to produce myokines, hepatokines, and adipokines. The imbalanced crosstalk between myokines, hepatokines, and adipokines may be harmful to the body; in consequence, it promotes the risk of various physiological disorders, such as obesity, type 2 diabetes mellitus (T2DM), metabolic syndrome, and cardiovascular disease (CVD).^35^ The crosstalk of liver with skeletal muscle and adipose tissue plays an essential role in pathological liver disease, such as nonalcoholic fatty liver disease (NAFLD) or metabolic dysfunction‐associated steatotic liver disease (MASLD). The progression of MASLD is significantly associated with the malfunction of skeletal muscle, which promotes the formation of myosteatosis, sarcopenic obesity, and sarcopenia.^36^ Myosteatosis is also correlated with liver steatosis in MASLD, due to ectopic fat accumulation in skeletal muscle, where excess lipids are disposed from adipose tissue.^37^ Our recent study has proved that hepatic *Dnaja3*‐deficienct riggers the onset of fatty liver, leading to mitochondrial dysfunction, fat accumulation, and concomitant disruption of fatty acid oxidation.^38^ As a result, we observed muscular *Dnaja3* heterozygosity mice elevated iWAT and eWAT accumulation in the whole body, speculating that may increase the risk of fatty liver disease, such as myosteatosis, during ageing (*Figure* 3B).

GMI, an effective drug to anti‐inflammation, has been widely used for anti‐cancerous treatment. GMI treatment inhibits tumour necrosis factor alpha (TNFα)‐mediated matrix metallopeptidase 9 (MMP‐9) expression and migration ability of various cancer cells, subsequently impeding tumourigenicity and inducing cell apoptosis in non‐small lung cancer cell lines^16^ ^[S5,S6]^. GMI is also found to exhibit anti‐inflammatory effect of muscular tissues. Previous study has reported that the transition of M1 to M2 macrophage releases the anti‐inflammatory cytokines to promote skeletal muscle differentiation.^39^ Our data identify that GMI can improve myogenesis, down‐regulate phosphorylated‐STAT3 activation (an inflammation marker), and systemic inflammation, and ameliorate muscular locomotion in *Dnaja3* heterozygosity mice via DNAJA3 activation under early initiation of short‐term GMI treatment (*Figure* 5). Moreover, we observed that long‐term GMI treatment alleviated the pathological symptoms, including fat accumulation, hyperglycaemia, and inflammation (*Figure* 6). Additionally, our study presented induced white adipose browning under long‐term GMI treatment (*Figure* 6D,E). Together, GMI treatment can ameliorate systemic inflammation and promote lipolysis and thermogenesis in white adipose tissues.

In summary, these findings facilitate the novel therapeutic strategies of GMI via activating DNAJA3 to promote mitochondrial homeostasis and subsequently to mediate muscle function enhancement, metabolic syndrome amelioration and sarcopenic obesity resistance.

## Conflict of interest

The authors declare that they have no conflict of interest in relation to this work to disclose.

## Funding

This study was supported by National Science and Technology Council of Taiwan (MOST 107‐2314‐B‐075‐012, MOST‐108‐2314‐B‐075‐046, NSTC 110‐2320‐B‐A49A‐526, and NSTC 112‐2314‐B‐A49‐018), Taipei Veterans General Hospital, Taiwan (V111C‐201), and grants from Ministry of Education, Higher Education of Taiwan SPROUT Project for Cancer Progression Research Center (111W31302) and Cancer and Immunology Research Center (112W31101 and 113W031101).

## Supporting information


**Figure S1.** Down‐regulation of the subunit on muscular mitochondrial electron transport chain young *HSA‐Dnaja3* ^*f/+*^ mice after exercise fatigue test. Mitochondrial fractionated proteins of gastrocnemius muscle were collected from 2‐month‐old WT and *HSA‐Dnaja3* ^*f/+*^ mice after exercise fatigue test. Then, the protein extracts of the mitochondrial fraction were subject to immunoblot assays with antibodies against DNAJA3, voltage‐dependent anion channel (VDAC) and oxidative phosphorylation complexes (OXPHOS); the bar graph data summarized the quantification of the difference between WT and *HSA‐Dnaja3* ^*f/+*^ mice (WT *n* = 2, *HSA‐Dnaja3* ^*f/+*^ *n* = 3). Statistical analyses were performed by unpaired *t*‐test, mean ± SEM. **P < 0.01 compared with the WT group.

**Figure S2.** Elevated intramuscular lipid droplet accumulation of primary myoblasts in young *HSA‐Dnaja3* ^*f/+*^ mice. The muscular primary crude cells were isolated from the hindlimbs of 6‐week‐old WT and *HSA‐Dnaja3* ^*f/+*^ mice, respectively (*n* = 6 per group). (A) The representative phase contrast images of muscular primary crude cells from the WT and the *HSA‐Dnaja3* ^*f/+*^ mice, stained with oil‐red‐O, were collected. The red arrows indicated the sites of lipid droplet accumulation. (B) Then, the bar graph summarized the quantification of oil‐red‐O staining between the muscular primary crude cells of WT and the *HSA‐Dnaja3* ^*f/+*^, was measured at 492 nm. Statistical analyses were performed by unpaired *t*‐test, mean ± SEM. *P < 0.05 compared with the WT group.

**Data S1.** Supplemental References.

**Table S1.** List of primers.
**Table S2.** List of primary antibodies.

**Figure S3.** **Long‐**term GMI treatment not associated with enhancing mitochondrial respiratory proteins. The 3‐month‐old *HSA‐Dnaja3* ^*f/+*^ mice were started intraperitoneal injection with placebo, 1.6 or 3.2 mg/kg of GMI treatment twice a week consecutively for six months. The mitochondrial proteins fraction extracted from *HSA‐Dnaja3* ^*f/+*^ mice treated with placebo (*n* = 3) or GMI, respectively (*n* = 5 per group). The immunoblotting assays against with DNAJA3, VDAC, and OXPHOS were analysed; the bar graph data summarized the quantification of the difference among *HSA‐Dnaja3* ^*f/+*^ mice treated with placebo or GMI. Statistical analyses were performed by one‐way ANOVA, mean ± SEM.

**Figure S4.** GMI enhancing mitochondrial function in WT differentiated primary myoblasts. (A) The representative phase contrast images of primary myoblasts isolated from hind limbs of 6‐week‐old WT mice, treated with or without 0.5 μg/ml GMI undergoing myogenesis, were collected at different time intervals (day 1 and 4). (B) The crude protein extracts were collected from WT differentiated primary myoblasts treated with or without GMI after inducing myogenesis day 1 and day 4, respectively. The immunoblotting assays against with DNAJA3, MyHC, p‐STAT3, t‐STAT3, and OXPHOS were analysed; the bar graph data summarized quantification of the difference between WT differentiated primary myoblasts treated with or without GMI (*n* = 3 per group). (C) Then, the oxygen consumption rate (OCR) of the induced myoblasts treated with or without GMI undergoing myogenesis (day 4) was further analysed by Seahorse XFe24 Extracellular Flux Analyser (*left* panel). Oligo, oligomycin; FCCP, carbonyl cyanide‐4‐(trifluoromethoxy) phenylhydrazone; AA, antimycin A. Real‐time quadruplicate readings and mitochondrial respiration rates of basal, maximal respiration, spare respiratory capacity and ATP production were collected (*right* panel) (mock *n* = 3, with GMI *n* = 4). (D) The confocal representative images of WT differentiated primary myoblasts (day 4) treated with or without GMI, stained with 5 μM MitoSOX Red and the nuclei counterstained with DAPI, were collected (scale bar, 20 μm). Statistical analyses were performed by unpaired *t*‐test, mean ± SEM. *P < 0.05, compared with the mock group.

**Data S2.** Supplemental Methods.
