# Supplementary figures and images for: Regimen on Dnaja3 haploinsufficiency mediated sarcopenic obesity with imbalanced mitochondrial homeostasis and lipid metabolism
Source: J Cachexia Sarcopenia Muscle. 2024 Aug 12;15(5):2013–29. doi: 10.1002/jcsm.13549 (PMC11446717; doi:10.1002/jcsm.13549)

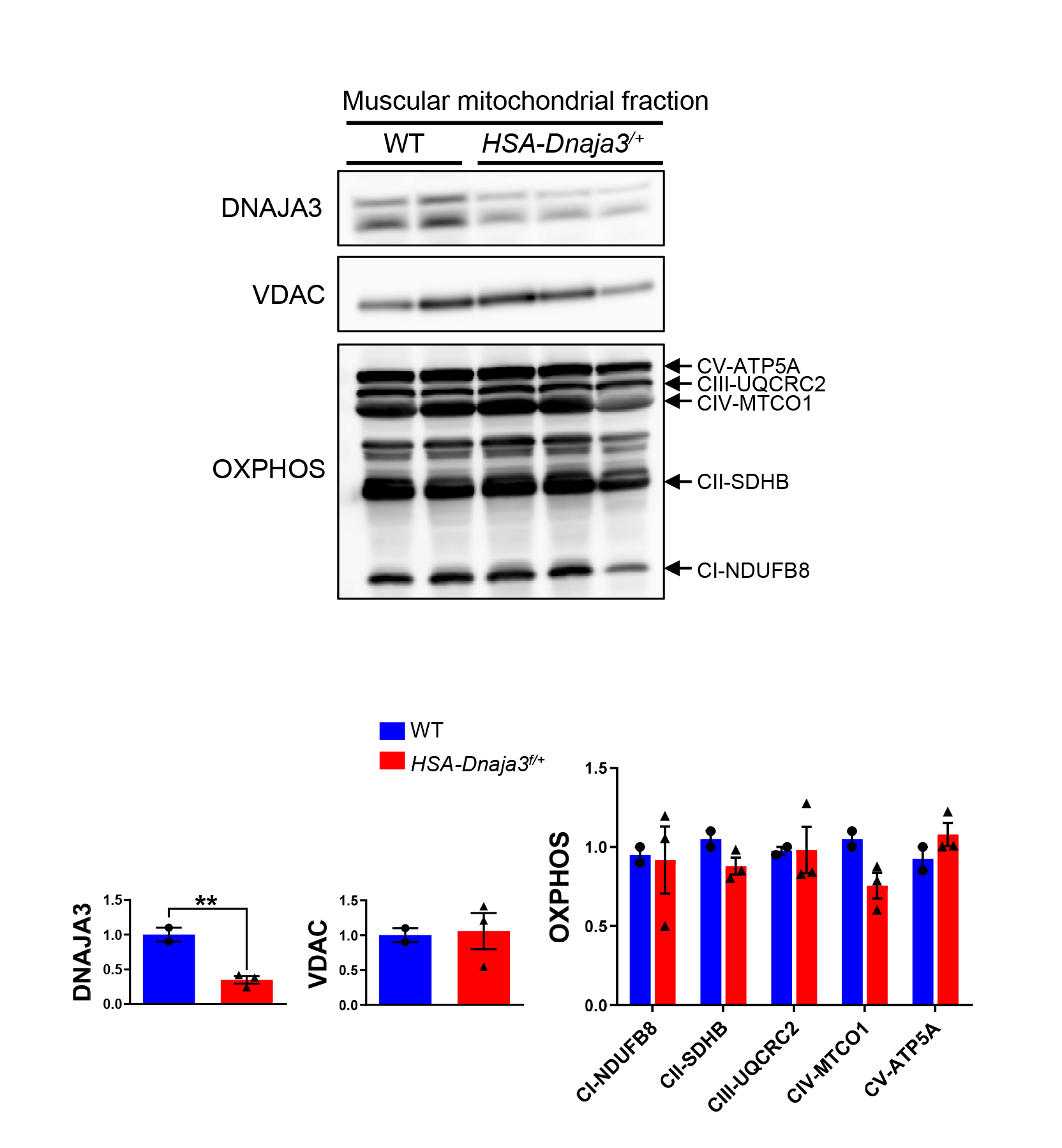

Supplement: Supplementary file 1 — Figure S1. Down‐regulation of the subunit on muscular mitochondrial electron transport chain young HSA‐Dnaja3 f/+ mice after exercise fatigue test. Mitochondrial fractionated proteins of gastrocnemius muscle were collected from 2‐month‐old WT and HSA‐Dnaja3 f/+ mice after exercise fatigue test. Then, the protein extracts of the mitochondrial fraction were subject to immunoblot assays with antibodies against DNAJA3, voltage‐dependent anion channel (VDAC) and oxidative phosphorylation complexes (OXPHOS); the bar graph data summarized the quantification of the difference between WT and HSA‐Dnaja3 f/+ mice (WT n = 2, HSA‐Dnaja3 f/+ n = 3). Statistical analyses were performed by unpaired t‐test, mean ± SEM. **P < 0.01 compared with the WT group. [file JCSM-15-2013-s005.tif]

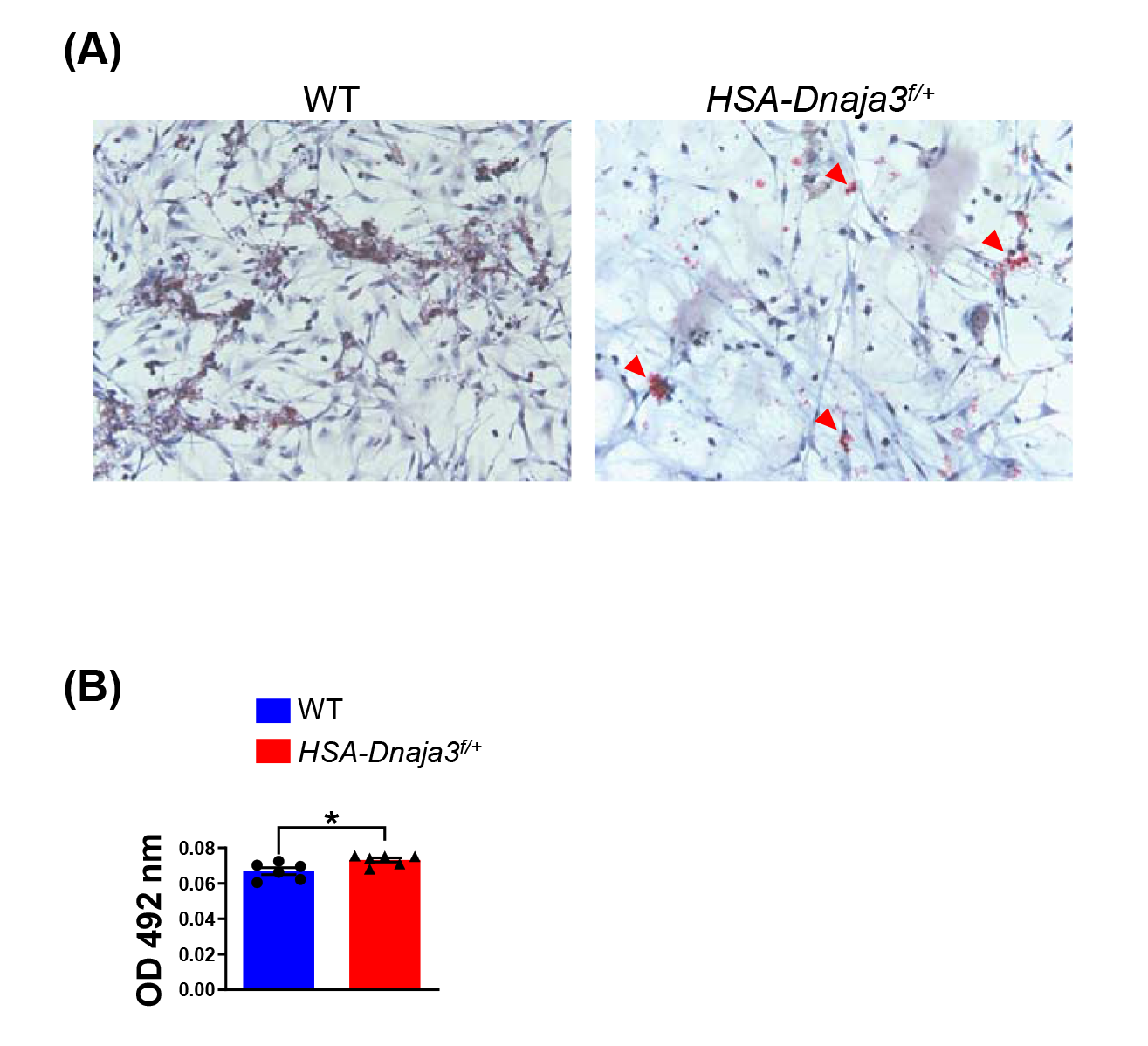

Supplement: Supplementary file 2 — Figure S2. Elevated intramuscular lipid droplet accumulation of primary myoblasts in young HSA‐Dnaja3 f/+ mice. The muscular primary crude cells were isolated from the hindlimbs of 6‐week‐old WT and HSA‐Dnaja3 f/+ mice, respectively (n = 6 per group). (A) The representative phase contrast images of muscular primary crude cells from the WT and the HSA‐Dnaja3 f/+ mice, stained with oil‐red‐O, were collected. The red arrows indicated the sites of lipid droplet accumulation. (B) Then, the bar graph summarized the quantification of oil‐red‐O staining between the muscular primary crude cells of WT and the HSA‐Dnaja3 f/+ , was measured at 492 nm. Statistical analyses were performed by unpaired t‐test, mean ± SEM. *P < 0.05 compared with the WT group. [file JCSM-15-2013-s007.tif]

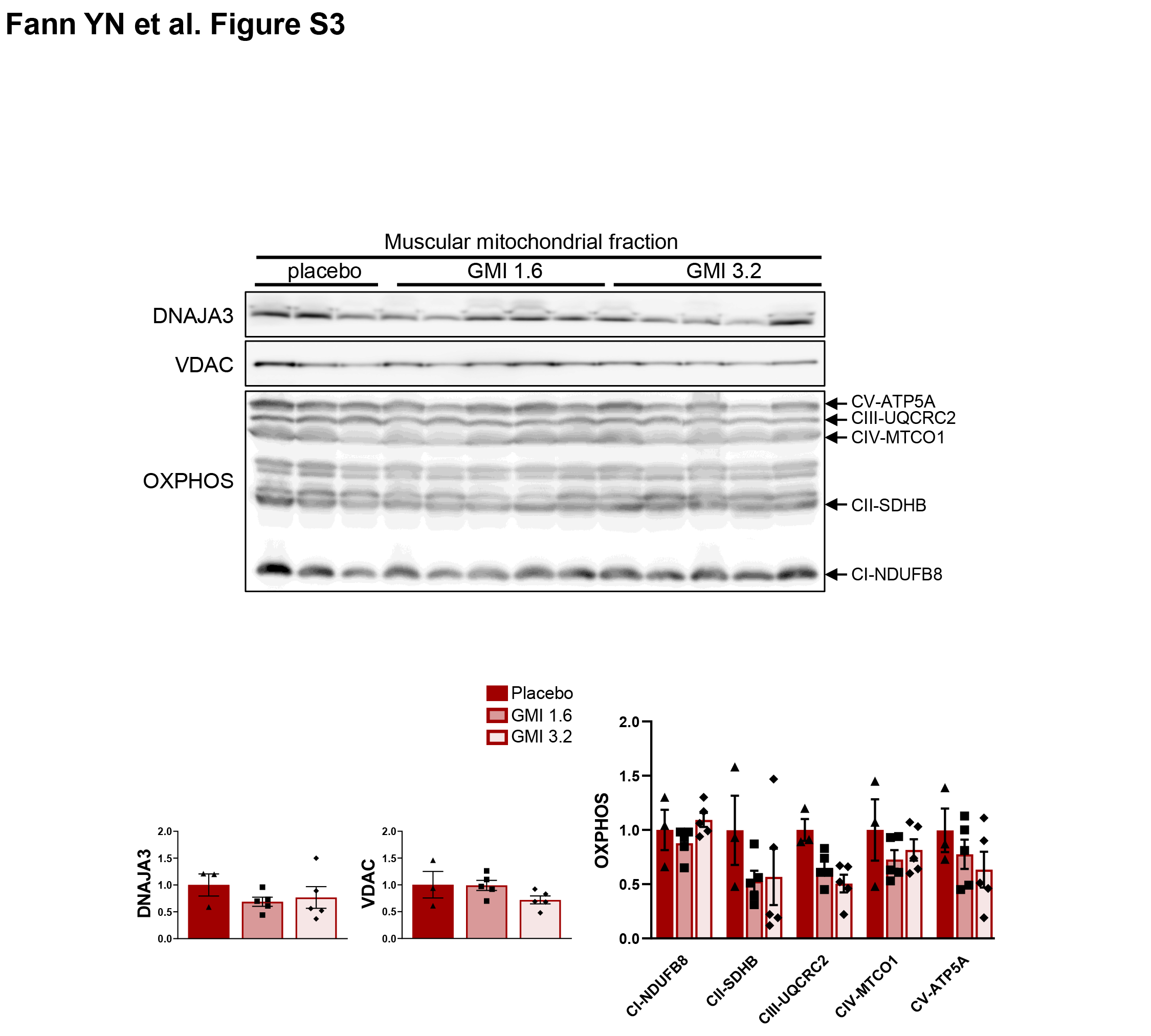

Supplement: Supplementary file 5 — Figure S3. Long‐term GMI treatment not associated with enhancing mitochondrial respiratory proteins. The 3‐month‐old HSA‐Dnaja3 f/+ mice were started intraperitoneal injection with placebo, 1.6 or 3.2 mg/kg of GMI treatment twice a week consecutively for six months. The mitochondrial proteins fraction extracted from HSA‐Dnaja3 f/+ mice treated with placebo (n = 3) or GMI, respectively (n = 5 per group). The immunoblotting assays against with DNAJA3, VDAC, and OXPHOS were analysed; the bar graph data summarized the quantification of the difference among HSA‐Dnaja3 f/+ mice treated with placebo or GMI. Statistical analyses were performed by one‐way ANOVA, mean ± SEM. [file JCSM-15-2013-s001.tif]

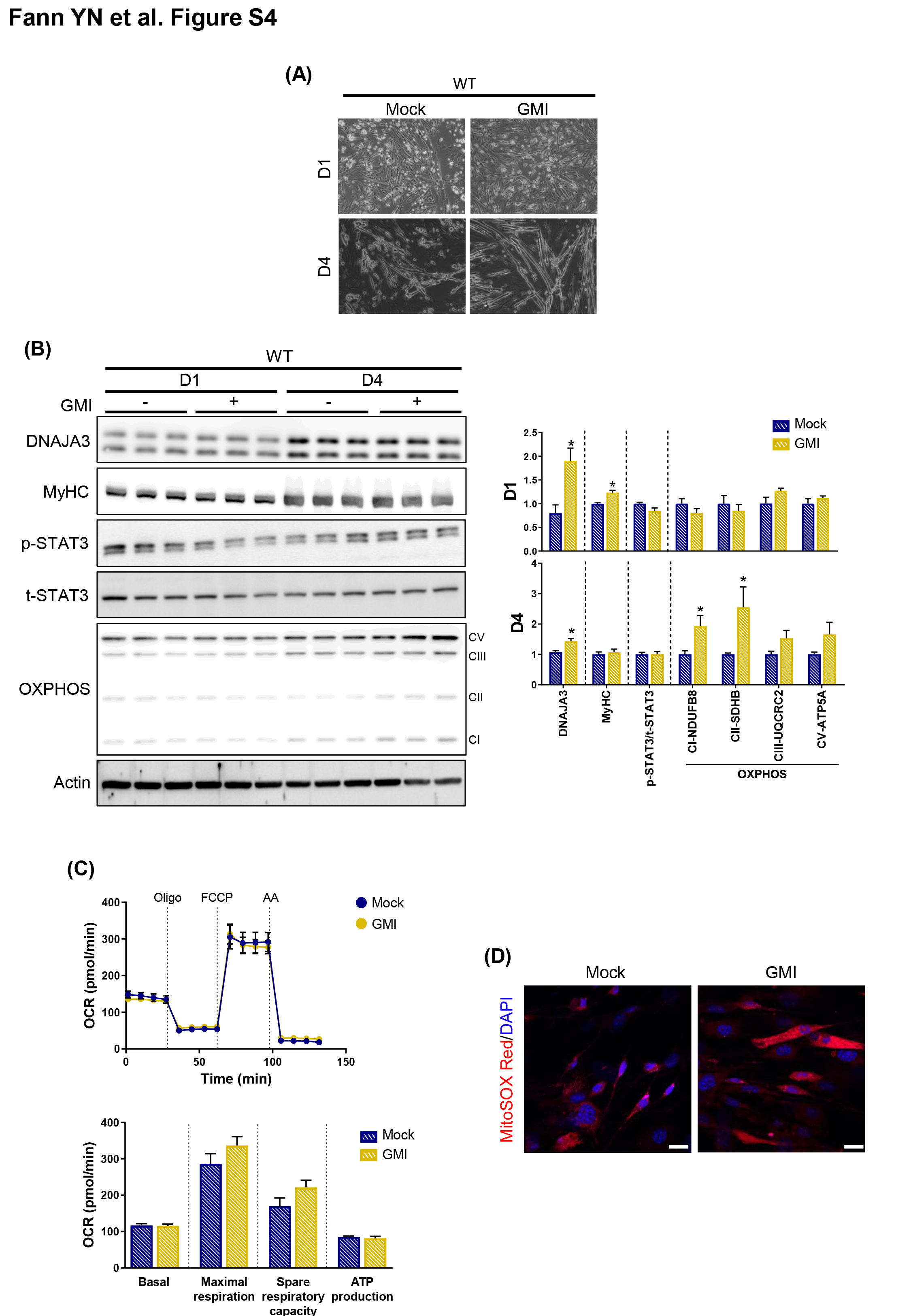

Supplement: Supplementary file 6 — Figure S4. GMI enhancing mitochondrial function in WT differentiated primary myoblasts. (A) The representative phase contrast images of primary myoblasts isolated from hind limbs of 6‐week‐old WT mice, treated with or without 0.5 μg/ml GMI undergoing myogenesis, were collected at different time intervals (day 1 and 4). (B) The crude protein extracts were collected from WT differentiated primary myoblasts treated with or without GMI after inducing myogenesis day 1 and day 4, respectively. The immunoblotting assays against with DNAJA3, MyHC, p‐STAT3, t‐STAT3, and OXPHOS were analysed; the bar graph data summarized quantification of the difference between WT differentiated primary myoblasts treated with or without GMI (n = 3 per group). (C) Then, the oxygen consumption rate (OCR) of the induced myoblasts treated with or without GMI undergoing myogenesis (day 4) was further analysed by Seahorse XFe24 Extracellular Flux Analyser (left panel). Oligo, oligomycin; FCCP, carbonyl cyanide‐4‐(trifluoromethoxy) phenylhydrazone; AA, antimycin A. Real‐time quadruplicate readings and mitochondrial respiration rates of basal, maximal respiration, spare respiratory capacity and ATP production were collected (right panel) (mock n = 3, with GMI n = 4). (D) The confocal representative images of WT differentiated primary myoblasts (day 4) treated with or without GMI, stained with 5 μM MitoSOX Red and the nuclei counterstained with DAPI, were collected (scale bar, 20 μm). Statistical analyses were performed by unpaired t‐test, mean ± SEM. *P < 0.05, compared with the mock group. [file JCSM-15-2013-s006.tif]
