# Supplementary material for: Regimen on Dnaja3 haploinsufficiency mediated sarcopenic obesity with imbalanced mitochondrial homeostasis and lipid metabolism
Source: J Cachexia Sarcopenia Muscle. 2024 Aug 12;15(5):2013–29. doi: 10.1002/jcsm.13549 (PMC11446717; doi:10.1002/jcsm.13549)
Supplement: Supplementary file 3 — Data S1. Supplemental References. [file JCSM-15-2013-s002.docx]

**Supplementary References**

**Regimen on *Dnaja3* Haploinsufficiency Mediated Sarcopenic Obesity with Imbalanced Mitochondrial Homeostasis and Lipid Metabolism**

*Journal of Cachexia, Sarcopenia and Muscle*

Yu-Ning Fann^1^, Wan-Huai Teo^2^, **Hsin-Chen Lee^1,3^**, Chen-Chung Liao**^4,5^**, Yeou-Guang Tsay**^6^**, Tung-Fu Huang**^7,8^***, Jeng-Fan Lo**^1,2,5,9,10^***

Affiliations:

^1^Institute of Pharmacology, College of Medicine, National Yang Ming Chiao Tung University, Taipei, Taiwan,

^2^Institute of Oral Biology, College of Dentistry, National Yang Ming Chiao Tung University, Taipei, Taiwan,

**^3^Department of Pharmacy, College of Pharmaceutical Sciences, National Yang Ming Chiao Tung University, Taipei, Taiwan,**

**^4^**Mass Spectrometry Facility, Instrumentation Resource Center, National Yang Ming Chiao Tung University, Taipei, Taiwan,

**^5^**Cancer Progression Research Center, National Yang Ming Chiao Tung University, Taipei, Taiwan,

**^6^**Institute of Biochemistry and Molecular Biology, College of Life Science, National Yang Ming Chiao Tung University, Taipei, Taiwan,

**^7^**School of Medicine, College of Medicine, National Yang Ming Chiao Tung University, Taipei, Taiwan,

**^8^**Department of Orthopedics and Traumatology, Taipei Veterans General Hospital, Taipei, Taiwan,

**^9^**Department of Dentistry, College of Dentistry, National Yang Ming Chiao Tung University, Taipei, Taiwan,

**^10^**Department of Dentistry, Taipei Veterans General Hospital, Taipei, Taiwan,

*Corresponding author

Jeng-Fan Lo,

Institute of Oral Biology, College of Dentistry, National Yang Ming Chiao Tung University, Taipei, 112304, Taiwan.

E-mail: jflo@nycu.edu.tw

and

Tung-Fu Huang,

Department of Orthopedics and Traumatology, Taipei Veterans General Hospital, No.201, Sec. 2, Shipai Rd., Taipei, 11217, Taiwan.

E-mail: huangtf@vghtpe.gov.tw

Supplementary References

S1. Kim J, Wang Z, Heymsfield SB, Baumgartner RN, Gallagher D. Total-body skeletal muscle mass: estimation by a new dual-energy X-ray absorptiometry method. Am J Clin Nutr. 2002;76:378-83.

S2. Janssen I, Heymsfield SB, Ross R. Low relative skeletal muscle mass (sarcopenia) in older persons is associated with functional impairment and physical disability. J Am Geriatr Soc. 2002;50:889-96.

S3. Marzetti E, Calvani R, Cesari M, Buford TW, Lorenzi M, Behnke BJ, et al. Mitochondrial dysfunction and sarcopenia of aging: from signaling pathways to clinical trials. Int J Biochem Cell Biol. 2013;45:2288-301.

S4. Akhmedov D, Berdeaux R. The effects of obesity on skeletal muscle regeneration. Front Physiol. 2013;4:371.

S5. Hsin IL, Ou CC, Wu TC, Jan MS, Wu MF, Chiu LY, et al. GMI, an immunomodulatory protein from Ganoderma microsporum, induces autophagy in non-small cell lung cancer cells. Autophagy. 2011;7:873-82.

S6. Hsin IL, Ou CC, Wu MF, Jan MS, Hsiao YM, Lin CH, et al. GMI, an Immunomodulatory Protein from Ganoderma microsporum, Potentiates Cisplatin-Induced Apoptosis via Autophagy in Lung Cancer Cells. Mol Pharm. 2015;12:1534-43.

S7. Walter L, Baruah A, Chang HW, Pace HM, Lee SS. The homeobox protein CEH-23 mediates prolonged longevity in response to impaired mitochondrial electron transport chain in C. elegans. PLoS Biol. 2011;9:e1001084.

S8. Kirchman PA, Kim S, Lai CY, Jazwinski SM. Interorganelle signaling is a determinant of longevity in Saccharomyces cerevisiae. Genetics. 1999;152:179-90.

S9. Glund S, Schoelch C, Thomas L, Niessen HG, Stiller D, Roth GJ, et al. Inhibition of acetyl-CoA carboxylase 2 enhances skeletal muscle fatty acid oxidation and improves whole-body glucose homeostasis in db/db mice. Diabetologia. 2012;55:2044-53.

S10. Rosa G, Manco M, Vega N, Greco AV, Castagneto M, Vidal H, et al. Decreased muscle acetyl-coenzyme A carboxylase 2 mRNA and insulin resistance in formerly obese subjects. Obes Res. 2003;11:1306-12.

S11. Choi CS, Savage DB, Abu-Elheiga L, Liu ZX, Kim S, Kulkarni A, et al. Continuous fat oxidation in acetyl-CoA carboxylase 2 knockout mice increases total energy expenditure, reduces fat mass, and improves insulin sensitivity. Proc Natl Acad Sci U S A. 2007;104:16480-5.

S12. Wettmarshausen J, Perocchi F. Isolation of Functional Mitochondria from Cultured Cells and Mouse Tissues. Methods Mol Biol. 2017;1567:15-32.

S13. Hindi L, McMillan JD, Afroze D, Hindi SM, Kumar A. Isolation, Culturing, and Differentiation of Primary Myoblasts from Skeletal Muscle of Adult Mice. Bio Protoc. 2017;7.

S14. Dougherty JP, Springer DA, Gershengorn MC. The Treadmill Fatigue Test: A Simple, High-throughput Assay of Fatigue-like Behavior for the Mouse. J Vis Exp. 2016.
