# Supplementary material for: Regimen on Dnaja3 haploinsufficiency mediated sarcopenic obesity with imbalanced mitochondrial homeostasis and lipid metabolism
Source: J Cachexia Sarcopenia Muscle. 2024 Aug 12;15(5):2013–29. doi: 10.1002/jcsm.13549 (PMC11446717; doi:10.1002/jcsm.13549)
Supplement: Supplementary file 4 — Table S1. List of primers. Table S2. List of primary antibodies. [file JCSM-15-2013-s003.docx]

**Supplementary Tables**

**Regimen on *Dnaja3* Haploinsufficiency Mediated Sarcopenic Obesity with Imbalanced Mitochondrial Homeostasis and Lipid Metabolism**

*Journal of Cachexia, Sarcopenia and Muscle*

Yu-Ning Fann^1^, Wan-Huai Teo^2^, **Hsin-Chen Lee^1,3^**, Chen-Chung Liao**^4,5^**, Yeou-Guang Tsay**^6^**, Tung-Fu Huang**^7,8^***, Jeng-Fan Lo**^1,2,5,9,10^***

Affiliations:

^1^Institute of Pharmacology, College of Medicine, National Yang Ming Chiao Tung University, Taipei, Taiwan,

^2^Institute of Oral Biology, College of Dentistry, National Yang Ming Chiao Tung University, Taipei, Taiwan,

**^3^Department of Pharmacy, College of Pharmaceutical Sciences, National Yang Ming Chiao Tung University, Taipei, Taiwan,**

**^4^**Mass Spectrometry Facility, Instrumentation Resource Center, National Yang Ming Chiao Tung University, Taipei, Taiwan,

**^5^**Cancer Progression Research Center, National Yang Ming Chiao Tung University, Taipei, Taiwan,

**^6^**Institute of Biochemistry and Molecular Biology, College of Life Science, National Yang Ming Chiao Tung University, Taipei, Taiwan,

**^7^**School of Medicine, College of Medicine, National Yang Ming Chiao Tung University, Taipei, Taiwan,

**^8^**Department of Orthopedics and Traumatology, Taipei Veterans General Hospital, Taipei, Taiwan,

**^9^**Department of Dentistry, College of Dentistry, National Yang Ming Chiao Tung University, Taipei, Taiwan,

**^10^**Department of Dentistry, Taipei Veterans General Hospital, Taipei, Taiwan,

*Corresponding author

Jeng-Fan Lo,

Institute of Oral Biology, College of Dentistry, National Yang Ming Chiao Tung University, Taipei, 112304, Taiwan.

E-mail: jflo@nycu.edu.tw

and

Tung-Fu Huang,

Department of Orthopedics and Traumatology, Taipei Veterans General Hospital, No.201, Sec. 2, Shipai Rd., Taipei, 11217, Taiwan.

E-mail: huangtf@vghtpe.gov.tw

**Supplementary Tables**

**Table S1: List of primers.**

| **Gene name** | **Forward primer** | **Reverse primer** |
| --- | --- | --- |
| *IJKL* | GTTTAAGGCCAGTTTGTCTCAAAAC | ACTTGACTAGCCCTTAGCATC |
| *HSA-Cre* | CCGGTCGATGCAACGAGTGAT | ACCAGAGTCATCCTTAGCGCC |

**Table S2: List of primary antibodies.**

| **Primary Antibodies** | **Supplier** | **Cat. No.** |
| --- | --- | --- |
| DNAJA3 | Santa Cruz | sc-18819 |
| IL-6 | Santa Cruz | sc-1265 |
| p-STAT3 (Tyr705) | Cell Signaling | 9145 |
| STAT3 | GeneTex | GTX104616 |
| p-AMPK (Thr172) | Cell Signaling | 2535 |
| AMPK | Cell Signaling | 2532 |
| ACC2  CPT1A  UCP1 | Cell Signaling  Cell Signaling  Cell Signaling | 3662  12252  14670 |
| MyHC | Merk Millipore | 05-716-I |
| VDAC | Cell Signaling | 4866 |
| OXPHOS | Abcam | ab110413 |
| β-actin | GeneTex | GTX109639 |
