# Supplementary material for: Regimen on Dnaja3 haploinsufficiency mediated sarcopenic obesity with imbalanced mitochondrial homeostasis and lipid metabolism
Source: J Cachexia Sarcopenia Muscle. 2024 Aug 12;15(5):2013–29. doi: 10.1002/jcsm.13549 (PMC11446717; doi:10.1002/jcsm.13549)
Supplement: Supplementary file 7 — Data S2. Supplemental Methods. [file JCSM-15-2013-s004.docx]

**Supplementary Methods**

**Regimen on *Dnaja3* Haploinsufficiency Mediated Sarcopenic Obesity with Imbalanced Mitochondrial Homeostasis and Lipid Metabolism**

*Journal of Cachexia, Sarcopenia and Muscle*

Yu-Ning Fann^1^, Wan-Huai Teo^2^, **Hsin-Chen Lee^1,3^**, Chen-Chung Liao**^4,5^**, Yeou-Guang Tsay**^6^**, Tung-Fu Huang**^7,8^***, Jeng-Fan Lo**^1,2,5,9,10^***

Affiliations:

^1^Institute of Pharmacology, College of Medicine, National Yang Ming Chiao Tung University, Taipei, Taiwan,

^2^Institute of Oral Biology, College of Dentistry, National Yang Ming Chiao Tung University, Taipei, Taiwan,

**^3^Department of Pharmacy, College of Pharmaceutical Sciences, National Yang Ming Chiao Tung University, Taipei, Taiwan,**

**^4^**Mass Spectrometry Facility, Instrumentation Resource Center, National Yang Ming Chiao Tung University, Taipei, Taiwan,

**^5^**Cancer Progression Research Center, National Yang Ming Chiao Tung University, Taipei, Taiwan,

**^6^**Institute of Biochemistry and Molecular Biology, College of Life Science, National Yang Ming Chiao Tung University, Taipei, Taiwan,

**^7^**School of Medicine, College of Medicine, National Yang Ming Chiao Tung University, Taipei, Taiwan,

**^8^**Department of Orthopedics and Traumatology, Taipei Veterans General Hospital, Taipei, Taiwan,

**^9^**Department of Dentistry, College of Dentistry, National Yang Ming Chiao Tung University, Taipei, Taiwan,

**^10^**Department of Dentistry, Taipei Veterans General Hospital, Taipei, Taiwan,

*Corresponding author

Jeng-Fan Lo,

Institute of Oral Biology, College of Dentistry, National Yang Ming Chiao Tung University, Taipei, 112304, Taiwan.

E-mail: jflo@nycu.edu.tw

and

Tung-Fu Huang,

Department of Orthopedics and Traumatology, Taipei Veterans General Hospital, No.201, Sec. 2, Shipai Rd., Taipei, 11217, Taiwan.

E-mail: huangtf@vghtpe.gov.tw

**Supplementary Methods**

**Muscular Mitochondria isolation**

The mice were scarified by cervical dislocation. The gastrocnemius muscles were collected, weighted out and placed in an ice cold beaker. The tissues were minced into small pieces (2–4 mm) and washed several times with IB1_M_(+) buffer (100 mM KCl, 50 mM Tris–HCl pH 7.4, 2 mM EDTA-KOH pH 7.4, fatty acid-free BSA). The IB1_M_(+) buffer was decanted; then, 1 ml of IB2_M_(++) (200 mM KCl, 100 mM Tris–HCl pH 7.4, 4 Mm EDTA-KOH pH 7.4, 10 mM MgCl_2_)/0.1 g of tissues were added and incubated for 3 min with stirring on ice. The tissue pieces were transferred to a tissue grinder and homogenized with three strokes at 500 rpm. The homogenate was transferred to a centrifuge tube, and centrifuged at 600 × g for 10 min at 4 °C. The supernatant was filtered through a cell strainer of 70 μm pore size and immediately centrifuged at 10,400 × g for 10 min at 4 °C. The pellet containing mitochondria was resuspended with IB1_M_(++) [S12].

**Isolation and cultivation of murine primary myoblast**

Primary myoblasts were isolated as described as previous study [17][S13]. To further induce myogenesis, the myoblast growth medium (MGM, F-10 media, 20% FBS, 1% PSA, and 10 ng/ml fibroblast growth factor) was replaced with differentiated medium consisting of DMEM, 2% horse serum (#16050-130, Thermo Fisher Scientific, New Zealand), and 1% PSA to induce myogenesis. During myogenesis, primary myoblasts were treated with or without 0.5 μg/ml GMI for 4 days and further analyzed by cellular respiration assay and immunoblotting.

**Serological analysis**

The serum was obtained from *HSA-Dnaja3^f/+^* or WT mice at 2- or 13-month-old aged. Serum biochemical studies, including analysis of total cholesterol (T-CHO), triglycerides (TG), alanine amino transferase (ALT), and aspartate transaminase (AST), were measured and evaluated using a DRI-CHEM NX600i (FUJIFILM, Tokyo, Japan).

**Histological analysis**

Gastrocnemius muscles were fixed in 4% formaldehyde for 24 h at room temperature, embedded in paraffin, cut into 8- or 10- μm-thick sections and mounted in slides. Muscle sections were stained with haematoxylin and eosin (H&E) and Picro-sirius red stain. **For each mouse, the muscle cross-sectional areas were analyzed with 200 myofibers.** Histological images were scanned by Zeiss Axioscan 7 (Carl Zeiss Microscopy GmbH, Germany). The cross-sectional areas **were measured by ZEISS ZEN Blue and the Sirius Red positive areas were quantified by Image Studio Lite Ver 5.2.**

**Cellular respiration assays**

The cellular oxygen consumption rate (OCR) was measured using the Seahorse XFp Extracellular Flux Analyzer (Agilent Technology) according to the manufacturer’s instruction. Primary myoblasts were isolated from the hind limbs of 1.5-month-old *HSA-Dnaja3^f/+^* or WT mice and each chamber was seeded 2 x 10^4^ cells on the Seahorse XFp Cell Culture Microplates to induce differentiation. In addition to basal respiration, maximal, and spare mitochondrial respiration rates and ATP production were determined by sequential additions with 2 μg/ml of oligomycin, 2 μg/ml of carbonyl cyanide-4-(trifluoromethoxy) phenylhydrazone (FCCP), 5 μg/ml of antimycin A (AA), respectively.

**MitoSOX Red stain**

Differentiated primary myoblasts were incubated with 5 μM MitoSOX Red (M36008, Invitrogen, USA) at 37 °C for 30 min and further stained with 4’,6-diamidino-2-phenylindole (DAPI) to stain nuclei at 37 °C for 3 min, washed three times with PBS and fixed in 4% (w/v) paraformaldehyde for 20 min. MitoSOX Red fluorescence was visualized by confocal microscopy (Zeiss LSM880, Carl Zeiss Microscopy GmbH, Germany).

**Oil red O (ORO) staining**

Primary cells were isolated from hindlimbs of 1.5-month-old *HSA-Dnaja3^f/+^* or WT mice, cells were seeded on 24-well plate for 72 h incubation and treated with 500 μM oleic acid for 24 hours to induce lipid accumulation. Then, cells were washed twice with PBS, fixed in 4% paraformaldehyde for 1 hour and stained with ORO for 1 h. Finally, ORO stain was extracted by 100% (v/v) isopropanol to quantitatively measure the density of the stain at 492 nm using Spark® Multimode Microplate Reader (Infinite 200 Pro, TECAN).

**Glucose tolerance test and insulin tolerance test**

For glucose tolerance test, 13-month-old *HSA-Dnaja3^f/+^* and WT mice were fasted overnight (16 h) and injected intraperitoneal with a glucose solution (1.5 g/kg body weight). Blood glucose concentration was measured before and 10, 20, 30, 60, 90 and 120 minutes after glucose injection. For insulin tolerance test, 13-month-old *HSA-Dnaja3^f/+^* and WT mice were morning-fasted for 6 h and injected intraperitoneal with insulin solution at 0.75 IU/kg body weight. Blood glucose concentration was monitored before and 10, 20, 30, 60, and 90 min after insulin injection. Blood samples were collected from tail tip using a glucose meter (Contour plus, Ascensia Diabetes Care).

**Immunoblotting analysis**

Frozen gastrocnemius muscles were homogenized in total lysis buffer (1 M Tris-HCl pH 6.8, and 1% SDS) and primary myoblasts were lysed in RIPA (1% Triton X-100, 0.5% sodium deoxycholate, 50 mM Tris-HCl pH 8.0, 150 mM NaCl, and 0.1% SDS) both containing protease inhibitors, 5mM NaF and 5mM Na_3_VO_4_. The homogenates or lysate were centrifuged at 13,000 × g for 20 min at 4°C. Subsequently, protein concentration of the supernatant fractions was determined by using Pierce BCA Protein Assay Kit (cat#23227, Thermo Scientific™). Proteins were separated by SDS-polyacrylamide gels and transferred to a nitrocellulose membrane. The blots were probed with primary antibodies and further detected by secondary antibodies (Supporting information). Proteins were detected by Chemiluminescent HRP substrate (WBKLS0500, EMD Millipore Corporation) and visualized by using ImageQuant™ LAS 4000 (GE Healthcare, USA). Protein quantification was performed by Image Studio Lite Ver 5.2.

**SDS-PAGE and In-gel digestion**

The protein samples were extracted from the muscular mitochondrial fraction of *HSA-Dnaja3^f/+^* or WT mice, respectively, and resolved by 10% SDS-PAGE. A total of 70 μg of each protein sample was adapted to the gel in triplicate. Further, the gel was stained with Coomassie Brilliant Blue G-250 (Bio-Rad, Hercules, CA, USA) after electrophoresis. Each gel lane was cut into pieces, and the pieces were repeatedly destained in a mixed 50% acetonitrile and 25 mM NH_4_HCO_3_ (1:1, v/v) solution. After being dried with Speed-Vac (Thermo Electron, Waltham, MA, USA), the gel pieces were incubated in 25 mM NH_4_HCO_3,_ including 1% β-mercaptoethanol for rehydration at room temperature in the dark for 30 min. For cysteine alkylation, each gel piece was added 5% 4-vinylpyridine in 25 mM NH_4_HCO_3_ and 50% acetonitrile (1:1, v/v) for 30 min. The pieces were washed in 25 mM NH_4_HCO_3_ and were dried by Speed-Vac for 40 min. Modified trypsin (Promega, Mannheim, Germany) in 25 mM NH_4_HCO_3_ was added to digest the proteins in gel overnight at 37 °C. Subsequently, tryptic peptides were extracted from the gel pieces by 25 mM NH_4_HCO_3_ for 20 min, dried with Speed-Vac, and stored at -20 °C for further analysis.

**Liquid chromatography (LC)-mass spectrometry (MS)/MS analysis and protein identification**

The tryptic peptides were dissolved in 20 μl formic acid (0.1%, v/v) and were analyzed by LC-MS/MS (NanoAcquity UP LC system, Waters, Manchester, UK) connected to a hybrid linear ion trap (LTQ-Orbitrap Elite; Thermo Scientific, San Jose, CA) mass spectrometer. The mobile phases comprised solvent A (0.1% formic acid in water) and solvent B (0.1% formic acid in acetonitrile). The peptides were desalted by the C18 trap column and then further chromatographed in C18 tip column with a linear gradient of 3%-40% solvent B for 90 min, 40%-95% solvent B for 2 min, and 5% solvent B for 10 min at a flow rate of 0.5 μl/min. The eluted peptides were ionized at a spray voltage of 2.33 kV and applied to the mass spectrometer. MS data was acquired in data-dependent acquisition (isolation width: 2 Da), and a full scan with high resolution (> 30,000 full widths at half maximum) was conducted in the m/z range of 200 to 1500. Subsequently, MS/MS scans were performed for the six most highly charged ions (2+ and 3+). The fragmented ions of each chosen precursor were generated through collision-induced dissociation using helium gas, employing a 35% collision energy.

The acquired MS raw data was analyzed using the Peaks 7.5 Studio software for proteomics (Bioinformatics Solutions, Waterloo, Canada), processing a search against the UniProt mouse protein database (containing 17,063 protein sequences; released on January 2021). The false discovery rate (FDR) was controlled at 1% for protein identifications. All the protein identifications were descanted with UniPort ID with the subsequent parameters: 50 ppm peptide mass tolerance and 0.8 Da fragment mass tolerance; precursor mass search type, monoisotopic; enzyme, trypsin; max missed cleavage, 2; nonspecific cleavage, 1; S-pyridyl ethylation, methionine oxidation, and carbamidomethylation; and variable PTMs per peptide, 3. In addition, all the MS spectral counts were normalized to the total spectral counts of a sample.

**Ingenuity Pathway Analysis**

Ingenuity Pathway Analysis (IPA; QIAGEN Inc.) was used to deduce global network functions of all differentially expressed proteins of the muscular mitochondrial proteome. Accession numbers of protein ID and expression fold change of the differentially expressed proteins were subject to IPA software to perform biological functions. The significance was calculated by using Fisher’s exact test (*p*-value < 0.05).
